# A new two-parameter Rayleigh distribution: Statistical properties, actuarial measures, regression analysis, and applications

**DOI:** 10.1016/j.heliyon.2024.e36775

**Published:** 2024-08-26

**Authors:** Ahmed M. Gemeay, Eslam Hussam, Ehab M. Almetwally

**Affiliations:** a Department of Mathematics, Faculty of Science, Tanta University, Tanta 31527, Egypt; b Department of Accounting, College of Business Administration in Hawtat Bani Tamim, Prince Sattam bin Abdulaziz University, Saudi Arabia; c Department of Mathematics, Faculty of Science, Helwan University, Cairo, Egypt; d Department of Mathematics and Statistics, Faculty of Science, Imam Mohammad Ibn Saud Islamic University (IMSIU), Riyadh 11432, Saudi Arabia; e Faculty of Business Administration, Delta University for Science and Technology, Gamasa 11152, Egypt

**Keywords:** Generalized distribution, Insurance, Estimation, Real data, Simulation, Regression

## Abstract

This paper presents a novel two-parameter distribution derived from the Rayleigh distribution, thoroughly investigating its essential mathematical properties. We employ estimation techniques to determine the proposed distribution's estimated parameters. Through extensive simulation studies, we analyze and evaluate the asymptotic behavior of the model estimators. Furthermore, we calculate various actuarial measures to highlight the practical utility of the proposed distribution in actuarial science. To further substantiate the applicability of our distribution, we perform a comprehensive regression analysis. The practical relevance of the proposed distribution is demonstrated by modeling a lifetime dataset from the insurance field, where it exhibits a superior fit compared to existing distributions. The findings suggest that the new distribution significantly improves modeling capabilities, making it a valuable tool for theoretical research and practical applications in fields requiring accurate lifetime data modeling.

## 1 Introduction

In recent years, statisticians have been more interested in constructing new generalized distributions to research and develop more of the following than baseline models. These generalized distributions open new paths for investigating real-world problems and provide more modeling versatility for asymmetric and complex random processes. Consequently, the body of academic literature has many different models. The traditional distributions as Pareto distribution, which is one of the most important names used in representing financial services sets of data, don't often offer a good fit for many application areas; the Weibull distribution, which defines the characteristics of small losses appropriately but is not an appropriate candidate model for describing the behavior of large losses; the distribution function of both the log-normal and the gamma distributions do not have closed form expressions, resulting in challenging calculations; and the distribution function of both the normal and the gamma distributions do. Comparatively, the Rayleigh distribution with a fixed hazard rate (HR) form is unsuitable for fitting experimental data with different HR shapes, including falling, bimodal distribution (inverted bathtub), increasing, and bathtub-shaped hazard rates. These types of hazard rates are typical in disciplines like dependability and engineering. As a result of this limitation, academics have been inspired to develop model extensions with increased flexibility.

The widely recognized probability distribution called Rayleigh (R) distribution is named after Lord Rayleigh (1880). Its single parameter characterizes it and has widespread applications in various fields, including reliability analysis, signal processing, and oceanography. It is one of the most useful and fundamental distributions of lifespans obtained from statistical literature. Recent research has focused on exploring its properties, developing novel applications, and proposing efficient estimation methods. Owing to its significance in a variety of domains, this model has been the subject of studies by various writers, including Merovci [26], Almongy et al. [8], Ateeq et al. [11], Ahmad et al. [4], Olayode [32], Vodă [47], Ahmed et al. [5], Roy [41], Park [33], and Sarti et al. [44].

The concept of weighted distributions originated from Fisher's study on how ascertainment methods impact frequency estimation. Building on Fisher [20] ideas, Rao [35], [36] recognized the necessity for a unifying framework and identified different sampling scenarios that could be effectively modeled using weighted distributions. Zelen [48] later applied weighted distributions in the context of cell kinetics and early disease detection, using them to represent length-biased sampling. Asok and Kanchan [31] introduced results on univariate and bivariate cases of some weighted distributions. Saghir et al. [42] briefly reviewed some weighted distributions. Other significant weighted distributions and their characteristics have also been covered by Badmus et al. [12], Dey et al. [17], Reyad et al. [39], Ahmad et al. [3], Rashwan [38], Salama et al. [43], and Almuqrin et al. [9]. Actuaries are very interested in modeling insurance data using heavy-tailed distributions. Moreover, the chance of an adverse outcome is a common interest for actuaries and risk managers. The insurance loss data's high tail distributions are ideal for estimating the degree of company risk. Because of the significance of these kinds of data, scholars and practitioners have put forth several probability models appropriate for modeling this kind of data. Several popular models deal with financial returns, file sizes on network servers, insurance loss data, etc. For more information about these models, see Hogg and Klugman [22], Afify et al. [1], Qi [34], and Teamah et al. [46].

Regression of distribution refers to a statistical approach that extends traditional regression analysis to model the relationships between distributions rather than just central tendencies like means or medians. This technique is particularly useful when the data exhibits significant variability or asymmetry or when understanding the entire distribution is more informative than summarizing it with a single metric. The best technique to make a regression model by distribution is quantile regression. Instead of estimating the mean, quantile regression estimates the conditional quantiles of the dependent variable. This provides a more comprehensive picture of the relationship, capturing the effects on different distribution points. For more information see [15], [40], [29], [7].

In this article, we are driven to design a more versatile alternative to the R model called the weighted Rayleigh (WDR) distribution, which may give better versatility in modeling insurance data. In addition, the article's purpose and motivation are

• Investigate a novel version of the R distribution based on the weighted-G (WD-G) family of distributions introduced by Bakouch et al. [13].
• Derive the fundamental statistical properties of the WDR distribution.
• It can accept the hazard rate function (hrf) shapes such as decreasing, increasing, and bathtub. Its probability density function (PDF) is unimodal, reversed-J shaped, left-skewed, and right-skewed.
• It has the flexibility for modeling insurance real data set than the other competing distributions.
• Eight distinct estimation methods are utilized to obtain the proposed model estimators.
• To establish a recommendation for choosing the optimal estimation approach for estimating the WDR parameters, we investigate and assess the efficacy of many estimators by conducting empirical simulations and basing our findings on those simulations.
• Different risk indicators are calculated for the WDR distribution. These indicators are the value at risk (VaR), the tail value at risk (TVaR), the tail-variance (TV), the tail-variance premium (TVP), and the expected shortfall (ES). These indicators play a crucial role in portfolio management, particularly in periods of confusion.
• It offers the versatility to estimate the coefficient parameter of the regression model.

The article consists of eight sections. In Section 2, the proposed model is derived. Its essential properties are determined in Section 3. In Section 4, the model parameters are derived using conventional estimating approaches, and the simulation analysis is conducted. In Section 5, four actuarial measures are examined. Section 6 is devoted to evaluating real-world data analysis. Section 7 contains the proposed model regression analysis. Finally, some of our paper results are presented in Section 8.

## 2 The WDR distribution

In 2020, Bakouch et al. [13] derived a new family of generalized distributions called the weighted general (WD-G) family. Its cumulative distribution function (CDF) is

(1) $$G(x)=\frac{\log[1+G^{a}(x)]}{\log(2)};\;x\in R,\;a>0.$$

The new statistical model is obtained by taking R distribution as a baseline distribution for the CDF (1), called the WDR distribution. Its CDF and PDF are

(2) $$F(x)=\frac{\log\left[{\left(1-e^{-\frac{x^{2}}{2b^{2}}}\right)}^{a}+1\right]}{\log(2)};\;\;\;a,b>0,\;\;x>0,$$

(3) $$f(x)=\frac{axe^{-\frac{x^{2}}{2b^{2}}}{\left(1-e^{-\frac{x^{2}}{2b^{2}}}\right)}^{a-1}}{b^{2}\log(2)\left[{\left(1-e^{-\frac{x^{2}}{2b^{2}}}\right)}^{a}+1\right]}.$$

The survival function, also known as SF, and the hrf are both provided by:

$$S(x)=1-\frac{\log\left[{\left(1-e^{-\frac{x^{2}}{2b^{2}}}\right)}^{a}+1\right]}{\log(2)},$$

$$h(x)=\frac{ax{\left(1-e^{-\frac{x^{2}}{2b^{2}}}\right)}^{a}}{b^{2}\left(e^{\frac{x^{2}}{2b^{2}}}-1\right)\left[{\left(1-e^{-\frac{x^{2}}{2b^{2}}}\right)}^{a}+1\right]\log\left[\frac{2}{{\left(1-e^{-\frac{x^{2}}{2b^{2}}}\right)}^{a}+1}\right]}.$$

**Figure 1:**
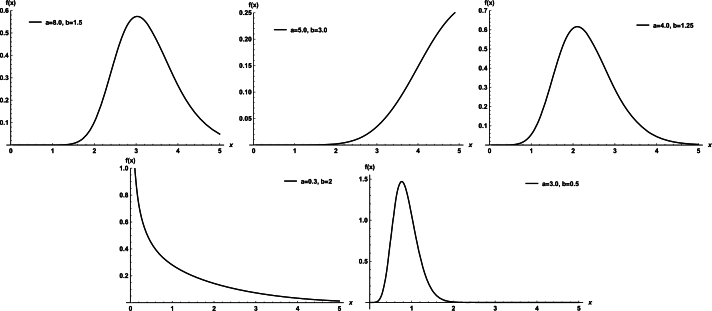
Plots of the WDR model PDF.

**Figure 2:**
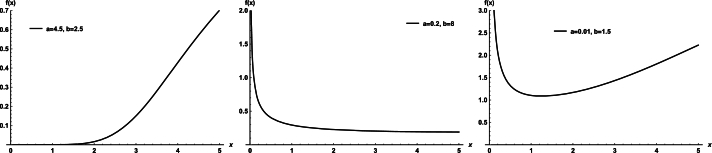
Plots of the WDR model hrf.

Plots of the WDR density for selected parameter values are shown in Fig. 1. Plots of the WDR hrf for selected parameter values are shown in Fig. 2. Its hrf is very flexible and can increase, decrease, and bathtub.

## 3 Statistical properties

This section discussed quantile function, linear representation, moments, incomplete moments, and order statistics for the WDR distribution.

### 3.1 Quantile function

Calculating WDR's quantile function (QF) is obtained by inverting the CDF (2) as follows

(4) $$Q(p)=b\sqrt{\log\left\{\frac{1}{{\left[1-{\left(2^{p}-1\right)}^{1/a}\right]}^{2}}\right\}},\;0<p<1.$$

By utilizing the QF (4), we can determine the proposed distribution's quartiles and produce random data sets of different sizes.

### 3.2 Linear representation

A helpful linear representation of the CDF and PDF of the WDR model is derived in this subsection. For −1<x≤1, we have

(5) $$\log(1+x)=\sum_{k=1}^{\infty }{\left(-1\right)}^{k+1}\frac{x^{k}}{k}.$$

Through the use of the power series (5) to (2). It is possible to express both the CDF and the PDF of the WDR distribution as

$$F(x)=\sum_{k=1}^{\infty }\Phi_{k}G_{k}(x),$$

$$f(x)=\sum_{k=1}^{\infty }\Phi_{k}g_{k}(x),$$

where $\Phi_{k}=\frac{{\left(-1\right)}^{k+1}}{k\log(2)}$ and $G_{k}(x)={\left(1-e^{-\frac{x^{2}}{2b^{2}}}\right)}^{ak}$ follows the exponentiated-Rayleigh (ExR) distribution with parameters *b*, and *ak*.

By making another expansion on F(x), we have

$$F(x)=\sum_{k=1}^{\infty }\sum_{m=0}^{\infty }\Phi_{k}{\left(-1\right)}^{m}\left(\begin{array}{c} a\;k\\ m \end{array}\right)e^{-\frac{mx^{2}}{2b^{2}}},$$

$$f(x)=\sum_{k=1}^{\infty }\sum_{m=0}^{\infty }\Phi_{k}{\left(-1\right)}^{m}\left(\begin{array}{c} a\;k\\ m \end{array}\right)\frac{mxe^{-\frac{mx^{2}}{2b^{2}}}}{b^{2}}=\sum_{k=1}^{\infty }\sum_{m=0}^{\infty }\Psi_{k,m}h_{m}(x),$$

where $\Psi_{k,m}=\frac{{\left(-1\right)}^{m+k+1}}{k\log(2)}\left(\begin{array}{c} ak\\ m \end{array}\right)$ and $h_{m}(x)=\frac{mxe^{-\frac{mx^{2}}{2b^{2}}}}{b^{2}}$ follows the R distribution with scale parameter $\frac{b}{\sqrt{m}}$.

### 3.3 Moments

The *r*th moments of the WDR distribution have the form

$$\mu_{r}^{\prime }=E(X^{r})=\int_{0}^{\infty }x^{r}f(x)dx=\sum_{k=1}^{\infty }\sum_{m=0}^{\infty }\Psi_{k,m}\int_{0}^{\infty }x^{r}h_{m}(x)dx=\sum_{k=1}^{\infty }\sum_{m=0}^{\infty }\Psi_{k,m}2^{r/2}\Gamma \left(\frac{r}{2}+1\right){\left(\frac{b}{\sqrt{m}}\right)}^{r}.$$

Setting r=1,2,3, and 4, respectively, we obtain the first four moments about the origin of the WDR distribution. Also, the WDR model moments used to have *n*th central moments and the cumulants ($k_{n}$) by the following relations

$$\mu_{n}=E{\left(x-\mu \right)}^{n}=\sum_{k=0}^{\infty }{\left(-1\right)}^{k}\left(\begin{array}{c} n\\ k \end{array}\right)\mu_{1}^{\prime k}\mu_{n-k}^{\prime }.$$

$$k_{n}=\mu_{n}^{\prime }-\sum_{k=0}^{n-1}\left(\begin{array}{c} n-1\\ k-1 \end{array}\right)k_{r}\mu_{n-r}^{\prime }.$$

The moment-generating function of the WDR distribution is represented in the following form

$$M(t)=\sum_{k=1}^{\infty }\sum_{m=0}^{\infty }\Psi_{k,m}\left\{\frac{\sqrt{\frac{\pi }{2}}bte^{\frac{b^{2}t^{2}}{2m}}\left[\text{erf}\left(\frac{bt}{\sqrt{2}\sqrt{m}}\right)+1\right]}{\sqrt{m}}+1\right\},\;\text{erf}(z)=\frac{2}{\sqrt{\pi }}\int_{0}^{z}e^{-t^{2}}dt.$$

The characteristic function of the WDR distribution is derived from the equation shown above by substituting *it* for the variable *t*.

### 3.4 Incomplete moments

The following expression describes the *s*th incomplete moment of the WDR distribution

$$\Psi_{s}(t)=\int_{0}^{t}x^{s}f(x)dx=\sum_{k=1}^{\infty }\sum_{m=0}^{\infty }\Psi_{k,m}2^{s/2}b^{s}m^{-\frac{s}{2}}\left[\Gamma \left(\frac{s}{2}+1\right)-\Gamma \left(\frac{s}{2}+1,\frac{mt^{2}}{2b^{2}}\right)\right],$$

where $\Gamma \left(\frac{s}{2}+1,\frac{mt^{2}}{2b^{2}}\right)$ is lower incomplete gamma function. The first incomplete moment calculates the mean residual life (MRL) and the mean waiting time. These two metrics are determined as follows $m_{1}(t)=[1-\Psi_{1}(t)]/S(t)-t$ and $M_{1}(t)=t-\Psi_{1}(t)/F(t)$.

### 3.5 Order statistics

Order statistics pertain to the statistical analysis of the values in a random sample arranged in ascending order. These statistics provide crucial insights into the properties and behavior of the sample, such as the minimum and maximum values, percentiles, and medians. The PDF and CDF of the *ith* order statistic for the WDR distribution are given by

$$f_{i:n}(x)=\frac{n!}{(i-1)!(n-i)!}{\left[F(x)\right]}^{i-1}{\left[1-F(x)\right]}^{n-i}f(x)=\frac{axn!e^{-\frac{x^{2}}{2b^{2}}}\log^{-i}(2){\left(1-e^{-\frac{x^{2}}{2b^{2}}}\right)}^{a-1}\log^{i-1}\left[{\left(1-e^{-\frac{x^{2}}{2b^{2}}}\right)}^{a}+1\right]{\left\{1-\frac{\log\left[{\left(1-e^{-\frac{x^{2}}{2b^{2}}}\right)}^{a}+1\right]}{\log(2)}\right\}}^{n-i}}{b^{2}\Gamma (i)\left[{\left(1-e^{-\frac{x^{2}}{2b^{2}}}\right)}^{a}+1\right]\Gamma (-i+n+1)},F_{i:n}(x)=\sum_{r=i}^{n}({}_{r}^{n}){\left(F(x)\right)}^{r}{\left(1-F(x)\right)}^{n-r}=\frac{\log^{-i}(2)\Gamma (n+1)\log^{i}\left[{\left(1-e^{-\frac{x^{2}}{2b^{2}}}\right)}^{a}+1\right]{\left\{1-\frac{\log\left[{\left(1-e^{-\frac{x^{2}}{2b^{2}}}\right)}^{a}+1\right]}{\log(2)}\right\}}^{n-i}H}{\Gamma (-i+n+1)},$$

where H=F˜12(1,i−n;i+1;log⁡(2)log⁡{12[(1−e−x22b2)a+1]}+1) is a hyper-geometric function.

## 4 Methods of estimation

In this part, we will explore eight different approaches to estimating the parameters of the WDR distribution represented by the equation $\theta ={\left(a,\;b\right)}^{⊤}$, and we will evaluate the behavior of these approaches using Monte Carlo simulations.

### 4.1 Methods

Suppose having a random order sample such as $x_{1},\ldots ,x_{n}$ having a size of *n* components coming from the PDF (3). In the case of *θ*, the log-likelihood function may be simplified to

$$\ell =(a-1)\sum_{i=1}^{n}\log\left(1-e^{-\frac{x_{i}^{2}}{2b^{2}}}\right)-\sum_{i=1}^{n}\log\left[\left(1-e^{-\frac{x_{i}^{2}}{2b^{2}}}\right){}^{a}+1\right]-\sum_{i=1}^{n}\frac{x_{i}^{2}}{2b^{2}}+\sum_{i=1}^{n}\log\left(x_{i}\right)+n\log\left[\frac{a}{b^{2}\log(2)}\right].$$

We achieve the maximum likelihood estimate (MLE) of *θ* by optimizing *ℓ* to its full potential.

Supposing that $x_{1:n},\ldots ,x_{n:n}$ is an order sample. First, one must minimize the function to calculate the parameters' ordinary least-squares estimates (OLSEs).

$$O=\sum_{i=1}^{n}{\left[F(x_{i:n})-\frac{i}{n+1}\right]}^{2}=\sum_{i=1}^{n}{\left[\frac{\log\left[{\left(1-e^{-\frac{x_{i:n}^{2}}{2b^{2}}}\right)}^{a}+1\right]}{\log(2)}-\frac{i}{n+1}\right]}^{2}.$$

The weighted least-squares estimators, often known as WLSEs, may be computed by minimizing the total number of squares for the following relation.

$$W=\sum_{i=1}^{n}\frac{{\left(n+1\right)}^{2}(n+2)}{i(n-i+1)}{\left\{F(x_{i:n})-\frac{i}{n+1}\right\}}^{2}=\sum_{i=1}^{n}\frac{{\left(n+1\right)}^{2}(n+2)}{i(n-i+1)}{\left\{\frac{\log\left[{\left(1-e^{-\frac{x_{i:n}^{2}}{2b^{2}}}\right)}^{a}+1\right]}{\log(2)}-\frac{i}{n+1}\right\}}^{2}.$$

In addition, the Anderson-Darling estimates (ADEs) are derived by reducing the following equation to its lowest possible value.

$$A=-n-\frac{1}{n}\sum_{i=1}^{n}(2i-1)\left[\log F(x_{i:n})+\log S(x_{i:n})\right],$$

the right tail Anderson-Darling estimates (RTADEs) of WDR distribution were obtained by minimizing the following relation.

$$R=\frac{n}{2}-2\sum_{i=1}^{n}F(x_{i:n})-\frac{1}{n}\sum_{i=1}^{n}(2i-1)\log S(x_{i:n}),$$

whereas the Cramér–von Mises estimates (CVMEs) are determined by minimizing.

$$CV=\frac{1}{12n}+\sum_{i=1}^{n}{\left[F(x_{i:n})-\frac{2i-1}{2n}\right]}^{2}=\frac{1}{12n}+\sum_{i=1}^{n}{\left\{\frac{\log\left[{\left(1-e^{-\frac{x_{i:n}^{2}}{2b^{2}}}\right)}^{a}+1\right]}{\log(2)}-\frac{2i-1}{2n}\right\}}^{2}.$$

The maximum product of spacing estimates (MPSEs) are derived from uniform spacings and maximize the following equation.

$$D_{i}=F(x_{i})-F(x_{i-1}),$$

where $F(x_{0})=0$, $F(x_{n+1}=1)$, $\sum_{i=1}^{n+1}D_{i}=1$, and they follow by maximizing

$$G=\frac{1}{n+1}\sum_{i=1}^{n+1}\log(D_{i}).$$

Lastly, the percentile estimates (PCEs) are determined by minimization of the given equation.

$$PCE=\sum_{i=1}^{n}{\left[x_{i:n}-Q(p_{i})\right]}^{2}=\sum_{i=1}^{n}{\left\{x_{i:n}-b\sqrt{\log\left(\frac{1}{{\left(1-{\left(2^{p_{i}}-1\right)}^{1/a}\right)}^{2}}\right)}\right\}}^{2},$$

where $p_{i}=i/(n+1)$ is an estimate of $F(x_{i:n})$.

### 4.2 The simulation design

Using the results of simulations, we investigate how well the estimate approaches that were just stated work when calculating WDR parameters. We will use the sample sizes n = 20, 70, 150, 300, and 500 and various parameter values. We create N=1,000 random samples from the WDR distribution by using the R software and compute the average absolute biases (ABBs), mean square errors (MSEs), mean relative estimates (MREs), average absolute difference between the theoretical and estimated CDFs ($D_{abs}$), and the maximum absolute difference between the theoretical and estimated CDFs ($D_{max}$).

They can be represented using the following equations.

$$ABBs=\frac{1}{N}\sum_{i=1}^{N}|\overset{ˆ}{\theta_{i}}-\theta_{i}|,\;\;\;\;MSEs=\frac{1}{N}\sum_{i=1}^{N}{\left(\overset{ˆ}{\theta_{i}}-\theta_{i}\right)}^{2},\;\;\;\;MREs=\frac{1}{N}\sum_{i=1}^{N}|\overset{ˆ}{\theta_{i}}-\theta_{i}|/\theta_{i},$$

$$D_{abs}=\frac{1}{n\;N}\sum_{i=1}^{N}\sum_{j=1}^{n}|F(x_{ij}|\theta_{i})-F(x_{ij}|\overset{ˆ}{\theta_{i}})|,\;\;D_{max}=\frac{1}{N}\sum_{i=1}^{N}\max_{j}|F(x_{ij}|\theta_{i})-F(x_{ij}|\overset{ˆ}{\theta_{i}})|,$$

where $\theta_{i}$ represents *a* and *b* parameters. Also, we compute the percentile bootstrap confidence interval length (CIL), coverage probability (CP), and average scales absolute error (ASAE) for all parameter combinations. Table 1, Table 2, Table 3, Table 4, Table 5 report the numerical values of our simulation, and Table 6 reports the ranks of each estimated method.

**Table 1:** Simulation values of the WDR model for (*a* = 0.25, *b* = 0.75).

n	Est.	Est. Par.	MLEs	ADEs	CVMEs	MPSEs	OLSEs	PCEs	RTADEs	WLSEs
20	AESTs	$\overset{ˆ}{a}$	0.27463	0.25588	0.28694	0.2408	0.25557	0.24653	0.27421	0.25735
		$\overset{ˆ}{b}$	0.69931	0.79637	0.73835	0.87382	0.86173	0.89892	0.76516	0.84007
	BIAS	$\overset{ˆ}{a}$	0.05149^{3}^	0.04709^{2}^	0.07335^{7}^	0.04476^{1}^	0.0536^{4}^	0.07905^{8}^	0.06177^{6}^	0.05519^{5}^
		$\overset{ˆ}{b}$	0.16383^{1}^	0.18789^{3}^	0.22038^{5}^	0.20746^{4}^	0.25041^{7}^	0.26744^{8}^	0.1803^{2}^	0.22453^{6}^
	MSE	$\overset{ˆ}{a}$	0.00579^{5}^	0.00373^{2}^	0.01877^{8}^	0.00352^{1}^	0.00505^{3}^	0.01034^{7}^	0.00798^{6}^	0.00551^{4}^
		$\overset{ˆ}{b}$	0.04074^{1}^	0.05924^{3}^	0.07895^{4}^	0.07967^{5}^	0.11201^{7}^	0.17576^{8}^	0.05483^{2}^	0.08362^{6}^
	MRE	$\overset{ˆ}{a}$	0.20595^{3}^	0.18835^{2}^	0.29339^{7}^	0.17903^{1}^	0.21439^{4}^	0.31622^{8}^	0.24708^{6}^	0.22075^{5}^
		$\overset{ˆ}{b}$	0.21843^{1}^	0.25052^{3}^	0.29384^{5}^	0.27661^{4}^	0.33389^{7}^	0.35659^{8}^	0.2404^{2}^	0.29937^{6}^
	CIL	$\overset{ˆ}{a}$	0.28152^{4}^	0.25585^{2}^	0.37751^{8}^	0.20506^{1}^	0.28656^{5}^	0.37058^{7}^	0.35445^{6}^	0.27742^{3}^
		$\overset{ˆ}{b}$	0.64927^{1}^	0.98259^{3}^	1.03572^{4}^	1.15587^{5}^	1.49081^{7}^	2.33568^{8}^	0.87318^{2}^	1.20627^{6}^
	CP	$\overset{ˆ}{a}$	0.884^{7}^	0.942^{2}^	0.85^{8}^	0.916^{4}^	0.948^{1}^	0.914^{5}^	0.892^{6}^	0.928^{3}^
		$\overset{ˆ}{b}$	0.832^{8}^	0.952^{2}^	0.846^{7}^	0.924^{5}^	0.956^{1}^	0.946^{3}^	0.91^{6}^	0.94^{4}^
	*D*_*abs*_		0.05388^{2}^	0.05521^{3}^	0.06419^{7}^	0.05292^{1}^	0.05993^{6}^	0.08559^{8}^	0.05764^{4}^	0.05856^{5}^
	*D*_*max*_		0.08648^{3}^	0.08618^{2}^	0.10664^{7}^	0.08184^{1}^	0.09414^{6}^	0.13398^{8}^	0.09196^{4}^	0.09291^{5}^
	*ASAE*		0.05014^{8}^	0.04289^{4}^	0.04419^{6}^	0.04349^{5}^	0.04451^{7}^	0.04035^{2}^	0.03892^{1}^	0.04053^{3}^
	∑*Ranks*		35^{1}^	43^{3}^	71^{6}^	38^{2}^	79^{7}^	90^{8}^	47^{4}^	65^{5}^

70	AESTs	$\overset{ˆ}{a}$	0.25543	0.25411	0.25678	0.24095	0.24819	0.24383	0.25479	0.25402
		$\overset{ˆ}{b}$	0.74261	0.75615	0.74757	0.80639	0.78458	0.80115	0.76221	0.76005
	BIAS	$\overset{ˆ}{a}$	0.02362^{1}^	0.02615^{4}^	0.02957^{7}^	0.02496^{2}^	0.02781^{5}^	0.03985^{8}^	0.02912^{6}^	0.02522^{3}^
		$\overset{ˆ}{b}$	0.0849^{1}^	0.09393^{2}^	0.11737^{7}^	0.10014^{5}^	0.11946^{8}^	0.11189^{6}^	0.0987^{3}^	0.1001^{4}^
	MSE	$\overset{ˆ}{a}$	0.00093^{2}^	0.00108^{3}^	0.00146^{7}^	9*e* − 04^{1}^	0.00127^{5}^	0.00245^{8}^	0.00145^{6}^	0.00109^{4}^
		$\overset{ˆ}{b}$	0.01094^{1}^	0.01453^{2}^	0.02205^{7}^	0.0166^{5}^	0.0228^{8}^	0.02086^{6}^	0.01602^{4}^	0.01538^{3}^
	MRE	$\overset{ˆ}{a}$	0.09448^{1}^	0.10459^{4}^	0.1183^{7}^	0.09983^{2}^	0.11125^{5}^	0.15939^{8}^	0.11649^{6}^	0.10089^{3}^
		$\overset{ˆ}{b}$	0.11319^{1}^	0.12524^{2}^	0.1565^{7}^	0.13353^{5}^	0.15928^{8}^	0.14919^{6}^	0.1316^{3}^	0.13347^{4}^
	CIL	$\overset{ˆ}{a}$	0.12096^{2}^	0.12386^{3}^	0.14331^{6}^	0.10683^{1}^	0.13371^{5}^	0.18879^{8}^	0.14467^{7}^	0.12723^{4}^
		$\overset{ˆ}{b}$	0.3867^{1}^	0.47081^{3}^	0.54275^{6}^	0.48122^{4}^	0.61244^{8}^	0.56383^{7}^	0.45161^{2}^	0.48869^{5}^
	CP	$\overset{ˆ}{a}$	0.912^{7}^	0.946^{1}^	0.924^{5}^	0.9^{8}^	0.924^{5}^	0.924^{5}^	0.93^{3}^	0.934^{2}^
		$\overset{ˆ}{b}$	0.9^{6}^	0.946^{2}^	0.896^{7}^	0.88^{8}^	0.958^{1}^	0.91^{5}^	0.914^{4}^	0.942^{3}^
	*D*_*abs*_		0.02734^{1}^	0.03037^{4}^	0.03277^{7}^	0.02927^{3}^	0.03243^{6}^	0.04066^{8}^	0.03151^{5}^	0.02921^{2}^
	*D*_*max*_		0.04346^{1}^	0.04788^{4}^	0.05339^{7}^	0.04561^{2}^	0.05161^{6}^	0.0641^{8}^	0.05021^{5}^	0.04659^{3}^
	*ASAE*		0.02116^{8}^	0.01891^{3}^	0.02053^{7}^	0.01985^{5}^	0.02007^{6}^	0.01822^{2}^	0.01791^{1}^	0.01931^{4}^
	∑*Ranks*		24^{1}^	48^{3}^	80^{6}^	44^{2}^	81^{7}^	82^{8}^	58^{5}^	51^{4}^

150	AESTs	$\overset{ˆ}{a}$	0.25401	0.2507	0.25354	0.24375	0.25175	0.24746	0.25252	0.25366
		$\overset{ˆ}{b}$	0.73842	0.75289	0.74777	0.77549	0.76354	0.77339	0.74857	0.74878
	BIAS	$\overset{ˆ}{a}$	0.01564^{2}^	0.01689^{3}^	0.01886^{5}^	0.01547^{1}^	0.01902^{6}^	0.02768^{8}^	0.02018^{7}^	0.01831^{4}^
		$\overset{ˆ}{b}$	0.05196^{1}^	0.06307^{4}^	0.07757^{7}^	0.06073^{2}^	0.07992^{8}^	0.06957^{6}^	0.06262^{3}^	0.06954^{5}^
	MSE	$\overset{ˆ}{a}$	0.00041^{2}^	0.00046^{3}^	0.00057^{5}^	0.00039^{1}^	0.00062^{6}^	0.00121^{8}^	0.00068^{7}^	0.00055^{4}^
		$\overset{ˆ}{b}$	0.00435^{1}^	0.00663^{4}^	0.00954^{7}^	0.0063^{3}^	0.00969^{8}^	0.00743^{5}^	0.00611^{2}^	0.00762^{6}^
	MRE	$\overset{ˆ}{a}$	0.06256^{2}^	0.06755^{3}^	0.07545^{5}^	0.06187^{1}^	0.07609^{6}^	0.11073^{8}^	0.0807^{7}^	0.07323^{4}^
		$\overset{ˆ}{b}$	0.06928^{1}^	0.0841^{4}^	0.10343^{7}^	0.08097^{2}^	0.10656^{8}^	0.09276^{6}^	0.08349^{3}^	0.09271^{5}^
	CIL	$\overset{ˆ}{a}$	0.08021^{2}^	0.08156^{3}^	0.09296^{6}^	0.07373^{1}^	0.09024^{5}^	0.13169^{8}^	0.09485^{7}^	0.08468^{4}^
		$\overset{ˆ}{b}$	0.26492^{1}^	0.31636^{4}^	0.37063^{7}^	0.3036^{3}^	0.39162^{8}^	0.34248^{6}^	0.30101^{2}^	0.32211^{5}^
	CP	$\overset{ˆ}{a}$	0.946^{1}^	0.942^{2}^	0.932^{3}^	0.894^{8}^	0.926^{6}^	0.928^{4.5}^	0.928^{4.5}^	0.924^{7}^
		$\overset{ˆ}{b}$	0.926^{4}^	0.942^{1.5}^	0.916^{8}^	0.922^{7}^	0.942^{1.5}^	0.924^{6}^	0.926^{4}^	0.926^{4}^
	*D*_*abs*_		0.01825^{1}^	0.01906^{3}^	0.02203^{6}^	0.01853^{2}^	0.02217^{7}^	0.02762^{8}^	0.02176^{5}^	0.0207^{4}^
	*D*_*max*_		0.02869^{1}^	0.03021^{3}^	0.03519^{6}^	0.02901^{2}^	0.03551^{7}^	0.04402^{8}^	0.03429^{5}^	0.03308^{4}^
	*ASAE*		0.0137^{8}^	0.01219^{4}^	0.0129^{6}^	0.01336^{7}^	0.01237^{5}^	0.01172^{2}^	0.01098^{1}^	0.01174^{3}^
	∑*Ranks*		35^{2}^	52.5^{3}^	74^{6}^	28^{1}^	84.5^{8}^	80.5^{7}^	58.5^{5}^	55^{4}^

300	AESTs	$\overset{ˆ}{a}$	0.25104	0.25004	0.25234	0.24768	0.25087	0.2472	0.25143	0.25074
		$\overset{ˆ}{b}$	0.74814	0.75273	0.74631	0.7655	0.75254	0.763	0.75171	0.75094
	BIAS	$\overset{ˆ}{a}$	0.01187^{4}^	0.01159^{2}^	0.01333^{5}^	0.0115^{1}^	0.01388^{7}^	0.01961^{8}^	0.01359^{6}^	0.01184^{3}^
		$\overset{ˆ}{b}$	0.04152^{1}^	0.04804^{6}^	0.05586^{7}^	0.04175^{2}^	0.05951^{8}^	0.0479^{5}^	0.04464^{3}^	0.04609^{4}^
	MSE	$\overset{ˆ}{a}$	0.00033^{7}^	0.00021^{1.5}^	0.00029^{4.5}^	0.00021^{1.5}^	3*e* − 04^{6}^	0.00059^{8}^	0.00029^{4.5}^	0.00023^{3}^
		$\overset{ˆ}{b}$	0.00369^{6}^	0.00362^{5}^	0.00487^{7}^	0.00305^{1}^	0.00566^{8}^	0.0036^{4}^	0.00314^{2}^	0.00326^{3}^
	MRE	$\overset{ˆ}{a}$	0.04747^{4}^	0.04637^{2}^	0.05333^{5}^	0.046^{1}^	0.05551^{7}^	0.07844^{8}^	0.05437^{6}^	0.04735^{3}^
		$\overset{ˆ}{b}$	0.05536^{1}^	0.06405^{6}^	0.07448^{7}^	0.05566^{2}^	0.07935^{8}^	0.06387^{5}^	0.05952^{3}^	0.06145^{4}^
	CIL	$\overset{ˆ}{a}$	0.05731^{3}^	0.0572^{2}^	0.06396^{6}^	0.05295^{1}^	0.0638^{5}^	0.09381^{8}^	0.06561^{7}^	0.05855^{4}^
		$\overset{ˆ}{b}$	0.20372^{1}^	0.2233^{4}^	0.26254^{7}^	0.20441^{2}^	0.26807^{8}^	0.23214^{6}^	0.21393^{3}^	0.22527^{5}^
	CP	$\overset{ˆ}{a}$	0.94^{4}^	0.948^{1}^	0.942^{3}^	0.938^{5}^	0.912^{8}^	0.932^{7}^	0.934^{6}^	0.944^{2}^
		$\overset{ˆ}{b}$	0.948^{2}^	0.942^{5}^	0.912^{6}^	0.946^{3}^	0.91^{7}^	0.906^{8}^	0.944^{4}^	0.95^{1}^
	*D*_*abs*_		0.014^{4}^	0.01361^{2}^	0.01523^{6}^	0.01275^{1}^	0.01559^{7}^	0.0194^{8}^	0.01478^{5}^	0.01374^{3}^
	*D*_*max*_		0.02244^{4}^	0.02176^{2}^	0.02447^{6}^	0.02032^{1}^	0.02548^{7}^	0.03078^{8}^	0.02343^{5}^	0.02181^{3}^
	*ASAE*		0.00901^{8}^	0.00799^{4}^	0.00821^{5}^	0.00881^{7}^	0.00857^{6}^	0.0075^{2}^	0.00727^{1}^	0.00797^{3}^
	∑*Ranks*		54^{5}^	48.5^{2}^	74.5^{7}^	30.5^{1}^	82^{8}^	72^{6}^	53.5^{4}^	53^{3}^
500	AESTs	$\overset{ˆ}{a}$	0.25072	0.24988	0.25049	0.24844	0.25082	0.24645	0.2507	0.25105
		$\overset{ˆ}{b}$	0.74823	0.75026	0.75158	0.76001	0.75032	0.76322	0.75147	0.74742
	BIAS	$\overset{ˆ}{a}$	0.00973^{3}^	0.00844^{2}^	0.01029^{5}^	0.00823^{1}^	0.01068^{7}^	0.01528^{8}^	0.01057^{6}^	0.00981^{4}^
		$\overset{ˆ}{b}$	0.03206^{1}^	0.03433^{3}^	0.04692^{8}^	0.03245^{2}^	0.0451^{7}^	0.03669^{6}^	0.03496^{4}^	0.03536^{5}^
	MSE	$\overset{ˆ}{a}$	0.00037^{8}^	0.00012^{1.5}^	0.00017^{4.5}^	0.00012^{1.5}^	0.00018^{6}^	0.00036^{7}^	0.00017^{4.5}^	0.00015^{3}^
		$\overset{ˆ}{b}$	0.00396^{8}^	0.00198^{3}^	0.00338^{7}^	0.00176^{1}^	0.00309^{6}^	0.00218^{5}^	0.00192^{2}^	0.00209^{4}^
	MRE	$\overset{ˆ}{a}$	0.03892^{3}^	0.03376^{2}^	0.04114^{5}^	0.03291^{1}^	0.04271^{7}^	0.06113^{8}^	0.04227^{6}^	0.03924^{4}^
		$\overset{ˆ}{b}$	0.04274^{1}^	0.04577^{3}^	0.06256^{8}^	0.04326^{2}^	0.06013^{7}^	0.04892^{6}^	0.04661^{4}^	0.04715^{5}^
	CIL	$\overset{ˆ}{a}$	0.04615^{4}^	0.04406^{2}^	0.04901^{6}^	0.04089^{1}^	0.04898^{5}^	0.07246^{8}^	0.05065^{7}^	0.04486^{3}^
		$\overset{ˆ}{b}$	0.16368^{2}^	0.17122^{4}^	0.20644^{8}^	0.15193^{1}^	0.20606^{7}^	0.17722^{6}^	0.16533^{3}^	0.17266^{5}^
	CP	$\overset{ˆ}{a}$	0.928^{5.5}^	0.934^{3}^	0.94^{2}^	0.928^{5.5}^	0.922^{7.5}^	0.93^{4}^	0.942^{1}^	0.922^{7.5}^
		$\overset{ˆ}{b}$	0.944^{3}^	0.948^{2}^	0.93^{4}^	0.97^{1}^	0.924^{7}^	0.906^{8}^	0.926^{6}^	0.928^{5}^
	*D*_*abs*_		0.01206^{5}^	0.01014^{2}^	0.01264^{7}^	0.00939^{1}^	0.01215^{6}^	0.01519^{8}^	0.01196^{4}^	0.01161^{3}^
	*D*_*max*_		0.01958^{5}^	0.01608^{2}^	0.02009^{7}^	0.01501^{1}^	0.01963^{6}^	0.02393^{8}^	0.01882^{4}^	0.01838^{3}^
	*ASAE*		0.00712^{8}^	0.00602^{4}^	0.00634^{6}^	0.00673^{7}^	0.00627^{5}^	0.00567^{2}^	0.00535^{1}^	0.00578^{3}^
	∑*Ranks*		57.5^{5}^	41.5^{2}^	83.5^{8}^	31^{1}^	72.5^{6}^	78^{7}^	56.5^{4}^	47.5^{3}^

**Table 2:** Simulation values of the WDR model for (*a* = 0.5, *b* = 2).

n	Est.	Est. Par.	MLEs	ADEs	CVMEs	MPSEs	OLSEs	PCEs	RTADEs	WLSEs
20	AESTs	$\overset{ˆ}{a}$	0.54885	0.52168	0.5643	0.46601	0.50969	0.50107	0.55334	0.52361
		$\overset{ˆ}{b}$	1.92805	2.02564	1.9749	2.28489	2.16154	2.21516	1.98705	2.08715
	BIAS	$\overset{ˆ}{a}$	0.10828^{3}^	0.10478^{2}^	0.1367^{8}^	0.10106^{1}^	0.11893^{4}^	0.12424^{6}^	0.13606^{7}^	0.12091^{5}^
		$\overset{ˆ}{b}$	0.31447^{1}^	0.35537^{2}^	0.42034^{5}^	0.44233^{7}^	0.47531^{8}^	0.43896^{6}^	0.36194^{3}^	0.4089^{4}^
	MSE	$\overset{ˆ}{a}$	0.02294^{3}^	0.02006^{2}^	0.04305^{8}^	0.01554^{1}^	0.02787^{5}^	0.02639^{4}^	0.04267^{7}^	0.03051^{6}^
		$\overset{ˆ}{b}$	0.15357^{1}^	0.19574^{2}^	0.2667^{5}^	0.30748^{6}^	0.37166^{7}^	0.37467^{8}^	0.21632^{3}^	0.26414^{4}^
	MRE	$\overset{ˆ}{a}$	0.21655^{3}^	0.20956^{2}^	0.27341^{8}^	0.20213^{1}^	0.23786^{4}^	0.24848^{6}^	0.27211^{7}^	0.24182^{5}^
		$\overset{ˆ}{b}$	0.15724^{1}^	0.17768^{2}^	0.21017^{5}^	0.22116^{7}^	0.23766^{8}^	0.21948^{6}^	0.18097^{3}^	0.20445^{4}^
	CIL	$\overset{ˆ}{a}$	0.64612^{5}^	0.58576^{2}^	0.88044^{8}^	0.42457^{1}^	0.65576^{6}^	0.62584^{3}^	0.85436^{7}^	0.64166^{4}^
		$\overset{ˆ}{b}$	1.39957^{1}^	1.77369^{3}^	1.89031^{4}^	2.20919^{6}^	2.41519^{7}^	2.49818^{8}^	1.68694^{2}^	2.05143^{5}^
	CP	$\overset{ˆ}{a}$	0.902^{6}^	0.942^{2}^	0.886^{7}^	0.874^{8}^	0.944^{1}^	0.92^{3}^	0.908^{5}^	0.918^{4}^
		$\overset{ˆ}{b}$	0.854^{8}^	0.932^{2}^	0.864^{7}^	0.908^{5}^	0.932^{2}^	0.912^{4}^	0.896^{6}^	0.932^{2}^
	*D*_*abs*_		0.05595^{1}^	0.05719^{2}^	0.05795^{4}^	0.0576^{3}^	0.06199^{7}^	0.06398^{8}^	0.05805^{5}^	0.06087^{6}^
	*D*_*max*_		0.08948^{1}^	0.08993^{3}^	0.09669^{5}^	0.08989^{2}^	0.09929^{7}^	0.10005^{8}^	0.09369^{4}^	0.09735^{6}^
	*ASAE*		0.04743^{8}^	0.04054^{3}^	0.04349^{6}^	0.04492^{7}^	0.04218^{5}^	0.04004^{1}^	0.04015^{2}^	0.04135^{4}^
	∑*Ranks*		32^{1}^	39^{2}^	70^{6}^	47^{3}^	83^{8}^	75^{7}^	57^{4}^	65^{5}^

70	AESTs	$\overset{ˆ}{a}$	0.51989	0.50684	0.51543	0.47944	0.50838	0.48791	0.51351	0.50604
		$\overset{ˆ}{b}$	1.97222	2.02109	2.00459	2.10893	2.03859	2.09941	2.00722	2.03731
	BIAS	$\overset{ˆ}{a}$	0.0575^{4}^	0.05333^{2}^	0.06288^{6}^	0.05309^{1}^	0.06065^{5}^	0.06983^{8}^	0.06763^{7}^	0.05562^{3}^
		$\overset{ˆ}{b}$	0.17908^{1}^	0.18628^{3}^	0.21276^{6}^	0.19423^{4}^	0.2243^{8}^	0.21367^{7}^	0.18537^{2}^	0.19744^{5}^
	MSE	$\overset{ˆ}{a}$	0.00554^{4}^	0.0048^{2}^	0.0067^{6}^	0.00426^{1}^	0.00662^{5}^	0.00777^{7}^	0.00814^{8}^	0.00499^{3}^
		$\overset{ˆ}{b}$	0.0478^{1}^	0.05717^{3}^	0.07323^{7}^	0.06132^{4}^	0.0804^{8}^	0.07127^{6}^	0.05574^{2}^	0.06359^{5}^
	MRE	$\overset{ˆ}{a}$	0.11501^{4}^	0.10666^{2}^	0.12576^{6}^	0.10618^{1}^	0.12129^{5}^	0.13966^{8}^	0.13527^{7}^	0.11125^{3}^
		$\overset{ˆ}{b}$	0.08954^{1}^	0.09314^{3}^	0.10638^{6}^	0.09712^{4}^	0.11215^{8}^	0.10683^{7}^	0.09269^{2}^	0.09872^{5}^
	CIL	$\overset{ˆ}{a}$	0.2699^{2}^	0.27118^{3}^	0.31848^{6}^	0.22551^{1}^	0.30024^{5}^	0.31929^{7}^	0.32596^{8}^	0.27772^{4}^
		$\overset{ˆ}{b}$	0.78953^{1}^	0.91221^{3}^	1.01921^{6}^	0.93758^{4}^	1.07659^{8}^	1.03375^{7}^	0.8956^{2}^	0.95249^{5}^
	CP	$\overset{ˆ}{a}$	0.886^{7}^	0.938^{2}^	0.914^{4}^	0.866^{8}^	0.928^{3}^	0.906^{6}^	0.91^{5}^	0.94^{1}^
		$\overset{ˆ}{b}$	0.904^{7}^	0.922^{4.5}^	0.936^{3}^	0.866^{8}^	0.942^{2}^	0.906^{6}^	0.922^{4.5}^	0.95^{1}^
	*D*_*abs*_		0.02994^{2}^	0.02925^{1}^	0.033^{6}^	0.03086^{4}^	0.03329^{7}^	0.03353^{8}^	0.03252^{5}^	0.03001^{3}^
	*D*_*max*_		0.04818^{3}^	0.04686^{1}^	0.05331^{6}^	0.04833^{4}^	0.05359^{7}^	0.0544^{8}^	0.05218^{5}^	0.04791^{2}^
	*ASAE*		0.02142^{8}^	0.01943^{4}^	0.01988^{6}^	0.02074^{7}^	0.01987^{5}^	0.01921^{2}^	0.01872^{1}^	0.0194^{3}^
	∑*Ranks*		35^{1}^	38.5^{3}^	78^{6}^	37^{2}^	84^{8}^	81^{7}^	57.5^{5}^	57^{4}^

150	AESTs	$\overset{ˆ}{a}$	0.50813	0.50239	0.51139	0.4873	0.504	0.48811	0.50321	0.50547
		$\overset{ˆ}{b}$	1.98326	2.00269	1.99401	2.07255	2.01873	2.04964	2.00574	2.00105
	BIAS	$\overset{ˆ}{a}$	0.03649^{1}^	0.03858^{3}^	0.04077^{5}^	0.03687^{2}^	0.04241^{6}^	0.04473^{8}^	0.04319^{7}^	0.03953^{4}^
		$\overset{ˆ}{b}$	0.11481^{1}^	0.13298^{4}^	0.15305^{8}^	0.13723^{6}^	0.14554^{7}^	0.12594^{2}^	0.1267^{3}^	0.13471^{5}^
	MSE	$\overset{ˆ}{a}$	0.00214^{2}^	0.00234^{3}^	0.00272^{5}^	0.00207^{1}^	0.00288^{6}^	0.00317^{8}^	0.00312^{7}^	0.00249^{4}^
		$\overset{ˆ}{b}$	0.02139^{1}^	0.02645^{4}^	0.03619^{8}^	0.02912^{6}^	0.03338^{7}^	0.02536^{3}^	0.02535^{2}^	0.02846^{5}^
	MRE	$\overset{ˆ}{a}$	0.07298^{1}^	0.07716^{3}^	0.08154^{5}^	0.07375^{2}^	0.08482^{6}^	0.08946^{8}^	0.08637^{7}^	0.07906^{4}^
		$\overset{ˆ}{b}$	0.0574^{1}^	0.06649^{4}^	0.07652^{8}^	0.06861^{6}^	0.07277^{7}^	0.06297^{2}^	0.06335^{3}^	0.06735^{5}^
	CIL	$\overset{ˆ}{a}$	0.1725^{2}^	0.17889^{3}^	0.20578^{6}^	0.1569^{1}^	0.19714^{5}^	0.22007^{8}^	0.20695^{7}^	0.18223^{4}^
		$\overset{ˆ}{b}$	0.54497^{1}^	0.61013^{4}^	0.68596^{7}^	0.6031^{2}^	0.70758^{8}^	0.65596^{6}^	0.60805^{3}^	0.61836^{5}^
	CP	$\overset{ˆ}{a}$	0.912^{7}^	0.928^{2.5}^	0.92^{5.5}^	0.872^{8}^	0.92^{5.5}^	0.928^{2.5}^	0.932^{1}^	0.926^{4}^
		$\overset{ˆ}{b}$	0.918^{5}^	0.93^{4}^	0.914^{7}^	0.888^{8}^	0.938^{1}^	0.932^{3}^	0.934^{2}^	0.916^{6}^
	*D*_*abs*_		0.01991^{1}^	0.02151^{4}^	0.02266^{8}^	0.02062^{2}^	0.02208^{7}^	0.022^{5}^	0.02205^{6}^	0.02089^{3}^
	*D*_*max*_		0.03182^{1}^	0.03428^{4}^	0.03644^{8}^	0.03292^{2}^	0.03544^{7}^	0.03534^{6}^	0.03529^{5}^	0.03382^{3}^
	*ASAE*		0.01322^{7}^	0.0125^{5}^	0.01289^{6}^	0.01357^{8}^	0.01247^{4}^	0.01233^{3}^	0.01161^{1}^	0.0123^{2}^
	∑*Ranks*		25^{1}^	52.5^{4}^	79.5^{7}^	40^{2}^	81.5^{8}^	71.5^{6}^	66^{5}^	52^{3}^

300	AESTs	$\overset{ˆ}{a}$	0.50305	0.49909	0.50434	0.49529	0.50282	0.49467	0.50145	0.50035
		$\overset{ˆ}{b}$	1.99157	2.00898	1.99138	2.03082	2.0051	2.03088	2.00529	2.00508
	BIAS	$\overset{ˆ}{a}$	0.02527^{2}^	0.02671^{4}^	0.0298^{5}^	0.02471^{1}^	0.03096^{7}^	0.03397^{8}^	0.03015^{6}^	0.02649^{3}^
		$\overset{ˆ}{b}$	0.08577^{1}^	0.08893^{4}^	0.10611^{8}^	0.08889^{3}^	0.10384^{7}^	0.09978^{6}^	0.08719^{2}^	0.09728^{5}^
	MSE	$\overset{ˆ}{a}$	0.00104^{2}^	0.00114^{4}^	0.00138^{5}^	0.00093^{1}^	0.00152^{7}^	0.00179^{8}^	0.00144^{6}^	0.0011^{3}^
		$\overset{ˆ}{b}$	0.01156^{1}^	0.01269^{4}^	0.01759^{8}^	0.01256^{3}^	0.01694^{7}^	0.01582^{6}^	0.01223^{2}^	0.01501^{5}^
	MRE	$\overset{ˆ}{a}$	0.05054^{2}^	0.05343^{4}^	0.0596^{5}^	0.04942^{1}^	0.06191^{7}^	0.06795^{8}^	0.0603^{6}^	0.05298^{3}^
		$\overset{ˆ}{b}$	0.04289^{1}^	0.04447^{4}^	0.05305^{8}^	0.04444^{3}^	0.05192^{7}^	0.04989^{6}^	0.0436^{2}^	0.04864^{5}^
	CIL	$\overset{ˆ}{a}$	0.11868^{2}^	0.12471^{3}^	0.13999^{6}^	0.11345^{1}^	0.13846^{5}^	0.15937^{8}^	0.1439^{7}^	0.12492^{4}^
		$\overset{ˆ}{b}$	0.38925^{1}^	0.42992^{4}^	0.48769^{7}^	0.40884^{2}^	0.49197^{8}^	0.45074^{6}^	0.42909^{3}^	0.43167^{5}^
	CP	$\overset{ˆ}{a}$	0.918^{7}^	0.93^{2}^	0.926^{4}^	0.924^{5.5}^	0.916^{8}^	0.924^{5.5}^	0.944^{1}^	0.928^{3}^
		$\overset{ˆ}{b}$	0.92^{5}^	0.928^{3}^	0.92^{5}^	0.884^{7}^	0.934^{2}^	0.88^{8}^	0.94^{1}^	0.92^{5}^
	*D*_*abs*_		0.01403^{2}^	0.01491^{3}^	0.01496^{4}^	0.01396^{1}^	0.01675^{8}^	0.0163^{7}^	0.01581^{6}^	0.01508^{5}^
	*D*_*max*_		0.02243^{2}^	0.02379^{3}^	0.02459^{5}^	0.02208^{1}^	0.02695^{8}^	0.02631^{7}^	0.02505^{6}^	0.02401^{4}^
	*ASAE*		0.00875^{7}^	0.00833^{4}^	0.00837^{5}^	0.00879^{8}^	0.00831^{3}^	0.00844^{6}^	0.00766^{1}^	0.0082^{2}^
	∑*Ranks*		29^{1}^	54^{3.5}^	75^{6}^	30.5^{2}^	82^{8}^	80.5^{7}^	63^{5}^	54^{3.5}^
500	AESTs	$\overset{ˆ}{a}$	0.5029	0.49855	0.50284	0.496	0.50188	0.49694	0.49911	0.50094
		$\overset{ˆ}{b}$	1.99229	2.01209	2.00117	2.02135	1.99958	2.01827	2.00946	2.00119
	BIAS	$\overset{ˆ}{a}$	0.01892^{1}^	0.01931^{2}^	0.02272^{5}^	0.01989^{3}^	0.02379^{7}^	0.02646^{8}^	0.02313^{6}^	0.01999^{4}^
		$\overset{ˆ}{b}$	0.06648^{1}^	0.06827^{2}^	0.07948^{7}^	0.07087^{5}^	0.08413^{8}^	0.0695^{4}^	0.0713^{6}^	0.06863^{3}^
	MSE	$\overset{ˆ}{a}$	0.00058^{1}^	0.00059^{2}^	0.00083^{6}^	0.00061^{3}^	0.00087^{7}^	0.00106^{8}^	0.00082^{5}^	0.00064^{4}^
		$\overset{ˆ}{b}$	0.00682^{1}^	0.00773^{4}^	0.01024^{7}^	0.00772^{3}^	0.01119^{8}^	0.00781^{5}^	0.00801^{6}^	0.00747^{2}^
	MRE	$\overset{ˆ}{a}$	0.03784^{1}^	0.03862^{2}^	0.04544^{5}^	0.03978^{3}^	0.04759^{7}^	0.05292^{8}^	0.04625^{6}^	0.03999^{4}^
		$\overset{ˆ}{b}$	0.03324^{1}^	0.03414^{2}^	0.03974^{7}^	0.03543^{5}^	0.04207^{8}^	0.03475^{4}^	0.03565^{6}^	0.03431^{3}^
	CIL	$\overset{ˆ}{a}$	0.09211^{2}^	0.09583^{3}^	0.10808^{6}^	0.08902^{1}^	0.10638^{5}^	0.12358^{8}^	0.1093^{7}^	0.09666^{4}^
		$\overset{ˆ}{b}$	0.29954^{1}^	0.3368^{5}^	0.37795^{7}^	0.31284^{2}^	0.37999^{8}^	0.34087^{6}^	0.33376^{3}^	0.3343^{4}^
	CP	$\overset{ˆ}{a}$	0.934^{4}^	0.952^{1}^	0.94^{3}^	0.92^{7}^	0.916^{8}^	0.93^{5.5}^	0.948^{2}^	0.93^{5.5}^
		$\overset{ˆ}{b}$	0.918^{6}^	0.938^{1}^	0.932^{3}^	0.91^{8}^	0.914^{7}^	0.928^{5}^	0.93^{4}^	0.934^{2}^
	*D*_*abs*_		0.01053^{1}^	0.01125^{4}^	0.01196^{5}^	0.01076^{2}^	0.01247^{7}^	0.01276^{8}^	0.01197^{6}^	0.01117^{3}^
	*D*_*max*_		0.01694^{1}^	0.01788^{4}^	0.01943^{6}^	0.01725^{2}^	0.02028^{7}^	0.02053^{8}^	0.01926^{5}^	0.0178^{3}^
	*ASAE*		0.00645^{7}^	0.00601^{2}^	0.00627^{5}^	0.00669^{8}^	0.00625^{4}^	0.00628^{6}^	0.00592^{1}^	0.00621^{3}^
	∑*Ranks*		26^{1}^	48^{4}^	78^{6}^	40^{2}^	79^{7}^	80.5^{8}^	69^{5}^	47.5^{3}^

**Table 3:** Simulation values of the WDR model for (*a* = 3, *b* = 1.5).

n	Est.	Est. Par.	MLEs	ADEs	CVMEs	MPSEs	OLSEs	PCEs	RTADEs	WLSEs
20	AESTs	$\overset{ˆ}{a}$	3.74261	3.42513	3.80957	2.69468	3.37746	2.79542	3.64352	3.22284
		$\overset{ˆ}{b}$	1.46188	1.5035	1.48269	1.62255	1.53492	1.59637	1.5118	1.54889
	BIAS	$\overset{ˆ}{a}$	1.17549^{5}^	1.06134^{3}^	1.40649^{8}^	0.90907^{2}^	1.24761^{6}^	0.83579^{1}^	1.40029^{7}^	1.07422^{4}^
		$\overset{ˆ}{b}$	0.15475^{2}^	0.15335^{1}^	0.17379^{4}^	0.20106^{8}^	0.18339^{7}^	0.17528^{5}^	0.16805^{3}^	0.17853^{6}^
	MSE	$\overset{ˆ}{a}$	3.38291^{4}^	2.73429^{3}^	8.14653^{8}^	1.74924^{2}^	5.3439^{6}^	1.103^{1}^	5.89534^{7}^	4.40794^{5}^
		$\overset{ˆ}{b}$	0.03716^{1}^	0.03823^{2}^	0.0484^{4}^	0.06622^{8}^	0.05347^{7}^	0.0492^{5}^	0.04698^{3}^	0.05046^{6}^
	MRE	$\overset{ˆ}{a}$	0.39183^{5}^	0.35378^{3}^	0.46883^{8}^	0.30302^{2}^	0.41587^{6}^	0.2786^{1}^	0.46676^{7}^	0.35807^{4}^
		$\overset{ˆ}{b}$	0.10317^{2}^	0.10223^{1}^	0.11586^{4}^	0.13404^{8}^	0.12226^{7}^	0.11685^{5}^	0.11203^{3}^	0.11902^{6}^
	CIL	$\overset{ˆ}{a}$	8.54252^{5}^	7.10449^{3}^	14.95358^{8}^	3.5899^{1}^	10.03837^{6}^	4.2344^{2}^	13.38025^{7}^	7.73753^{4}^
		$\overset{ˆ}{b}$	0.65923^{1}^	0.75613^{2}^	0.83563^{3}^	0.90063^{6}^	0.93194^{8}^	0.9064^{7}^	0.83826^{4}^	0.86987^{5}^
	CP	$\overset{ˆ}{a}$	0.856^{7}^	0.924^{3}^	0.902^{6}^	0.818^{8}^	0.946^{1}^	0.91^{5}^	0.912^{4}^	0.932^{2}^
		$\overset{ˆ}{b}$	0.858^{7}^	0.924^{3.5}^	0.9^{5}^	0.842^{8}^	0.946^{1}^	0.886^{6}^	0.924^{3.5}^	0.934^{2}^
	*D*_*abs*_		0.05424^{1}^	0.05521^{2}^	0.06056^{7}^	0.05999^{6}^	0.06225^{8}^	0.05785^{4}^	0.05905^{5}^	0.05547^{3}^
	*D*_*max*_		0.08866^{1}^	0.08883^{2}^	0.09908^{7}^	0.09472^{5}^	0.09993^{8}^	0.09052^{4}^	0.09674^{6}^	0.08906^{3}^
	*ASAE*		0.04665^{8}^	0.04204^{3}^	0.04269^{7}^	0.04253^{6}^	0.04252^{5}^	0.04125^{2}^	0.04232^{4}^	0.04036^{1}^
	∑*Ranks*		42^{2}^	39.5^{1}^	70^{7}^	59^{4}^	85^{8}^	47^{3}^	61.5^{5}^	64^{6}^

70	AESTs	$\overset{ˆ}{a}$	3.16081	3.12114	3.18634	2.84856	2.98012	2.86209	3.18852	3.133
		$\overset{ˆ}{b}$	1.49368	1.49881	1.49248	1.54701	1.52003	1.54235	1.49922	1.50177
	BIAS	$\overset{ˆ}{a}$	0.46863^{1}^	0.50792^{4}^	0.59335^{7}^	0.47508^{2}^	0.51476^{6}^	0.50357^{3}^	0.63591^{8}^	0.51311^{5}^
		$\overset{ˆ}{b}$	0.08109^{1}^	0.08515^{3}^	0.09726^{8}^	0.09038^{4}^	0.0918^{5}^	0.09323^{6}^	0.09612^{7}^	0.08126^{2}^
	MSE	$\overset{ˆ}{a}$	0.38885^{3}^	0.45147^{5}^	0.63576^{7}^	0.34009^{1}^	0.42805^{4}^	0.37932^{2}^	0.76728^{8}^	0.46906^{6}^
		$\overset{ˆ}{b}$	0.01034^{1}^	0.01215^{3}^	0.0142^{7}^	0.01302^{4}^	0.01335^{5}^	0.0136^{6}^	0.01452^{8}^	0.01062^{2}^
	MRE	$\overset{ˆ}{a}$	0.15621^{1}^	0.16931^{4}^	0.19778^{7}^	0.15836^{2}^	0.17159^{6}^	0.16786^{3}^	0.21197^{8}^	0.17104^{5}^
		$\overset{ˆ}{b}$	0.05406^{1}^	0.05677^{3}^	0.06484^{8}^	0.06025^{4}^	0.0612^{5}^	0.06215^{6}^	0.06408^{7}^	0.05417^{2}^
	CIL	$\overset{ˆ}{a}$	2.48462^{3}^	2.55013^{4}^	3.21801^{7}^	1.8687^{1}^	2.73625^{6}^	2.12909^{2}^	3.33165^{8}^	2.66996^{5}^
		$\overset{ˆ}{b}$	0.36586^{1}^	0.39631^{2}^	0.44142^{7}^	0.40882^{4}^	0.46118^{8}^	0.43119^{5}^	0.4321^{6}^	0.40666^{3}^
	CP	$\overset{ˆ}{a}$	0.9^{6}^	0.924^{4.5}^	0.926^{3}^	0.828^{8}^	0.946^{1}^	0.868^{7}^	0.924^{4.5}^	0.932^{2}^
		$\overset{ˆ}{b}$	0.91^{5}^	0.924^{4}^	0.928^{3}^	0.844^{8}^	0.946^{1}^	0.892^{7}^	0.904^{6}^	0.944^{2}^
	*D*_*abs*_		0.0297^{2}^	0.0307^{5}^	0.03265^{7}^	0.03067^{4}^	0.0327^{8}^	0.02961^{1}^	0.03178^{6}^	0.0301^{3}^
	*D*_*max*_		0.04817^{1.5}^	0.04968^{5}^	0.05375^{8}^	0.04941^{4}^	0.05267^{6}^	0.04817^{1.5}^	0.05313^{7}^	0.04896^{3}^
	*ASAE*		0.02066^{8}^	0.01885^{1}^	0.01961^{5}^	0.02005^{6}^	0.01958^{4}^	0.01934^{3}^	0.0201^{7}^	0.01891^{2}^
	∑*Ranks*		30.5^{1}^	48.5^{4}^	90^{8}^	38^{2}^	79^{6}^	42.5^{3}^	87.5^{7}^	52^{5}^

150	AESTs	$\overset{ˆ}{a}$	3.08993	3.05562	3.09603	2.8866	3.00424	2.94665	3.0584	3.01999
		$\overset{ˆ}{b}$	1.49619	1.50265	1.49749	1.52547	1.50741	1.51633	1.50255	1.50525
	BIAS	$\overset{ˆ}{a}$	0.32609^{3}^	0.31251^{2}^	0.39484^{8}^	0.29182^{1}^	0.35799^{6}^	0.33477^{5}^	0.37867^{7}^	0.32663^{4}^
		$\overset{ˆ}{b}$	0.05616^{3}^	0.05659^{4}^	0.06624^{8}^	0.05542^{1}^	0.06269^{7}^	0.06052^{5}^	0.06059^{6}^	0.05544^{2}^
	MSE	$\overset{ˆ}{a}$	0.18226^{5}^	0.16325^{2}^	0.27617^{8}^	0.13149^{1}^	0.20566^{6}^	0.17481^{4}^	0.25294^{7}^	0.16923^{3}^
		$\overset{ˆ}{b}$	0.00509^{3}^	0.00512^{4}^	0.0069^{8}^	0.00487^{2}^	0.00618^{7}^	0.00554^{5}^	0.00579^{6}^	0.00486^{1}^
	MRE	$\overset{ˆ}{a}$	0.1087^{3}^	0.10417^{2}^	0.13161^{8}^	0.09727^{1}^	0.11933^{6}^	0.11159^{5}^	0.12622^{7}^	0.10888^{4}^
		$\overset{ˆ}{b}$	0.03744^{3}^	0.03773^{4}^	0.04416^{8}^	0.03695^{1}^	0.0418^{7}^	0.04035^{5}^	0.0404^{6}^	0.03696^{2}^
	CIL	$\overset{ˆ}{a}$	1.52697^{3}^	1.6175^{4}^	1.92822^{7}^	1.30242^{1}^	1.80392^{6}^	1.49919^{2}^	1.99792^{8}^	1.61993^{5}^
		$\overset{ˆ}{b}$	0.25179^{1}^	0.27182^{3}^	0.29903^{7}^	0.26589^{2}^	0.30588^{8}^	0.2822^{5}^	0.29374^{6}^	0.27495^{4}^
	CP	$\overset{ˆ}{a}$	0.896^{7}^	0.952^{2}^	0.904^{6}^	0.88^{8}^	0.93^{3}^	0.908^{5}^	0.924^{4}^	0.954^{1}^
		$\overset{ˆ}{b}$	0.914^{6.5}^	0.934^{4}^	0.924^{5}^	0.904^{8}^	0.938^{2}^	0.914^{6.5}^	0.936^{3}^	0.946^{1}^
	*D*_*abs*_		0.02051^{2}^	0.02068^{3}^	0.023^{7}^	0.0193^{1}^	0.02167^{6}^	0.02069^{4}^	0.02314^{8}^	0.02144^{5}^
	*D*_*max*_		0.03339^{3}^	0.03309^{2}^	0.03761^{8}^	0.03102^{1}^	0.03523^{6}^	0.03353^{4}^	0.03754^{7}^	0.03447^{5}^
	*ASAE*		0.01271^{7}^	0.01165^{1}^	0.01218^{3}^	0.01275^{8}^	0.01243^{4}^	0.01246^{6}^	0.01244^{5}^	0.01202^{2}^
	∑*Ranks*		40.5^{2}^	43^{3}^	87^{8}^	22^{1}^	82^{6}^	56.5^{5}^	84^{7}^	53^{4}^

300	AESTs	$\overset{ˆ}{a}$	3.041	3.00592	3.05205	2.93122	2.99079	2.95737	3.0069	3.0221
		$\overset{ˆ}{b}$	1.49814	1.50329	1.49691	1.51362	1.50405	1.51323	1.50224	1.5
	BIAS	$\overset{ˆ}{a}$	0.21644^{2}^	0.21852^{3}^	0.27492^{8}^	0.19795^{1}^	0.25979^{6}^	0.23308^{5}^	0.27188^{7}^	0.22765^{4}^
		$\overset{ˆ}{b}$	0.03563^{1}^	0.03873^{3}^	0.04423^{6}^	0.03866^{2}^	0.04578^{8}^	0.04371^{5}^	0.04444^{7}^	0.04167^{4}^
	MSE	$\overset{ˆ}{a}$	0.07258^{2}^	0.07326^{3}^	0.12722^{8}^	0.06738^{1}^	0.11179^{6}^	0.08544^{4}^	0.12093^{7}^	0.08768^{5}^
		$\overset{ˆ}{b}$	0.00199^{1}^	0.00237^{2}^	0.00304^{7}^	0.00245^{3}^	0.0034^{8}^	0.00301^{5.5}^	0.00301^{5.5}^	0.00275^{4}^
	MRE	$\overset{ˆ}{a}$	0.07215^{2}^	0.07284^{3}^	0.09164^{8}^	0.06598^{1}^	0.0866^{6}^	0.07769^{5}^	0.09063^{7}^	0.07588^{4}^
		$\overset{ˆ}{b}$	0.02375^{1}^	0.02582^{3}^	0.02948^{6}^	0.02577^{2}^	0.03052^{8}^	0.02914^{5}^	0.02962^{7}^	0.02778^{4}^
	CIL	$\overset{ˆ}{a}$	1.0266^{2}^	1.08761^{4}^	1.29533^{7}^	0.94345^{1}^	1.2329^{6}^	1.06417^{3}^	1.33028^{8}^	1.11558^{5}^
		$\overset{ˆ}{b}$	0.17741^{1}^	0.19056^{3}^	0.21273^{8}^	0.18344^{2}^	0.21228^{7}^	0.19594^{5}^	0.20791^{6}^	0.19259^{4}^
	CP	$\overset{ˆ}{a}$	0.926^{4}^	0.948^{1}^	0.932^{3}^	0.898^{8}^	0.922^{5}^	0.916^{7}^	0.918^{6}^	0.934^{2}^
		$\overset{ˆ}{b}$	0.952^{2}^	0.96^{1}^	0.934^{3.5}^	0.916^{6}^	0.916^{6}^	0.906^{8}^	0.934^{3.5}^	0.916^{6}^
	*D*_*abs*_		0.01438^{1}^	0.01488^{3}^	0.01537^{6}^	0.0146^{2}^	0.01601^{8}^	0.01517^{5}^	0.01575^{7}^	0.01494^{4}^
	*D*_*max*_		0.02305^{1.5}^	0.02383^{3}^	0.02529^{6}^	0.02305^{1.5}^	0.02598^{8}^	0.02447^{5}^	0.02585^{7}^	0.02411^{4}^
	*ASAE*		0.00833^{7.5}^	0.00795^{2}^	0.00833^{7.5}^	0.00827^{6}^	0.00825^{5}^	0.00824^{4}^	0.00809^{3}^	0.00786^{1}^
	∑*Ranks*		34^{2}^	48^{3}^	89^{8}^	26.5^{1}^	83^{7}^	54.5^{5}^	80^{6}^	53^{4}^
500	AESTs	$\overset{ˆ}{a}$	3.02343	2.99329	3.01738	2.95375	3.03621	2.95904	3.02449	3.01135
		$\overset{ˆ}{b}$	1.49777	1.50111	1.50044	1.50957	1.49782	1.50713	1.49947	1.50081
	BIAS	$\overset{ˆ}{a}$	0.17497^{3}^	0.17562^{4}^	0.20924^{7}^	0.15096^{1}^	0.21558^{8}^	0.18282^{5}^	0.20608^{6}^	0.16831^{2}^
		$\overset{ˆ}{b}$	0.03035^{3}^	0.03174^{4}^	0.03456^{7}^	0.02966^{1}^	0.03751^{8}^	0.03204^{5}^	0.0336^{6}^	0.03018^{2}^
	MSE	$\overset{ˆ}{a}$	0.04809^{3}^	0.04872^{4}^	0.07086^{7}^	0.04014^{1}^	0.07582^{8}^	0.05081^{5}^	0.06586^{6}^	0.04393^{2}^
		$\overset{ˆ}{b}$	0.00146^{2}^	0.00156^{4}^	0.00191^{7}^	0.00142^{1}^	0.00221^{8}^	0.00159^{5}^	0.00174^{6}^	0.00147^{3}^
	MRE	$\overset{ˆ}{a}$	0.05832^{3}^	0.05854^{4}^	0.06975^{7}^	0.05032^{1}^	0.07186^{8}^	0.06094^{5}^	0.06869^{6}^	0.0561^{2}^
		$\overset{ˆ}{b}$	0.02024^{3}^	0.02116^{4}^	0.02304^{7}^	0.01977^{1}^	0.02501^{8}^	0.02136^{5}^	0.0224^{6}^	0.02012^{2}^
	CIL	$\overset{ˆ}{a}$	0.7893^{2}^	0.83178^{4}^	0.97104^{7}^	0.74225^{1}^	0.96205^{6}^	0.82378^{3}^	1.01676^{8}^	0.84952^{5}^
		$\overset{ˆ}{b}$	0.13855^{1}^	0.14836^{3}^	0.16388^{8}^	0.14104^{2}^	0.16373^{7}^	0.14965^{5}^	0.16119^{6}^	0.14862^{4}^
	CP	$\overset{ˆ}{a}$	0.916^{5.5}^	0.924^{4}^	0.926^{3}^	0.91^{8}^	0.916^{5.5}^	0.912^{7}^	0.936^{2}^	0.948^{1}^
		$\overset{ˆ}{b}$	0.92^{5.5}^	0.926^{4}^	0.93^{3}^	0.906^{8}^	0.914^{7}^	0.92^{5.5}^	0.956^{1}^	0.944^{2}^
	*D*_*abs*_		0.01137^{4}^	0.01169^{5}^	0.01188^{6}^	0.0107^{1}^	0.01205^{7}^	0.01108^{2}^	0.01247^{8}^	0.01126^{3}^
	*D*_*max*_		0.01834^{4}^	0.01876^{5}^	0.01953^{6}^	0.01712^{1}^	0.01975^{7}^	0.01815^{2}^	0.02014^{8}^	0.01817^{3}^
	*ASAE*		0.00608^{6}^	0.00589^{2}^	0.00612^{7}^	0.00615^{8}^	0.00606^{5}^	0.00605^{4}^	0.00595^{3}^	0.00583^{1}^
	∑*Ranks*		41^{2}^	53^{5}^	88^{8}^	21^{1}^	85.5^{7}^	51.5^{4}^	84^{6}^	44^{3}^

**Table 4:** Simulation values of the WDR model for (*a* = 2, *b* = 0.25).

n	Est.	Est. Par.	MLEs	ADEs	CVMEs	MPSEs	OLSEs	PCEs	RTADEs	WLSEs
20	AESTs	$\overset{ˆ}{a}$	2.3453	2.24186	2.40152	1.82681	2.16133	1.92627	2.40083	2.08293
		$\overset{ˆ}{b}$	0.24402	0.24941	0.24715	0.26974	0.25798	0.26667	0.25206	0.25908
	BIAS	$\overset{ˆ}{a}$	0.64604^{5}^	0.63068^{4}^	0.80815^{7}^	0.52343^{1}^	0.68799^{6}^	0.53105^{2}^	0.87728^{8}^	0.62093^{3}^
		$\overset{ˆ}{b}$	0.02691^{1}^	0.0279^{2}^	0.03112^{3}^	0.0327^{7}^	0.03361^{8}^	0.03248^{6}^	0.03229^{5}^	0.03226^{4}^
	MSE	$\overset{ˆ}{a}$	1.3149^{6}^	0.95446^{4}^	1.72288^{7}^	0.45161^{1}^	1.16531^{5}^	0.53379^{2}^	2.71967^{8}^	0.85371^{3}^
		$\overset{ˆ}{b}$	0.00114^{1}^	0.00122^{2}^	0.00148^{3}^	0.00167^{6}^	0.00186^{8}^	0.00179^{7}^	0.00163^{4}^	0.00165^{5}^
	MRE	$\overset{ˆ}{a}$	0.32302^{5}^	0.31534^{4}^	0.40407^{7}^	0.26172^{1}^	0.34399^{6}^	0.26553^{2}^	0.43864^{8}^	0.31047^{3}^
		$\overset{ˆ}{b}$	0.10763^{1}^	0.11162^{2}^	0.12448^{3}^	0.13082^{7}^	0.13446^{8}^	0.12993^{6}^	0.12915^{5}^	0.12902^{4}^
	CIL	$\overset{ˆ}{a}$	4.39955^{5}^	3.88724^{3}^	6.98582^{7}^	2.17347^{1}^	4.48193^{6}^	2.70285^{2}^	7.44006^{8}^	3.92618^{4}^
		$\overset{ˆ}{b}$	0.11947^{1}^	0.13716^{2}^	0.1499^{3}^	0.16165^{6}^	0.17161^{8}^	0.16747^{7}^	0.15091^{4}^	0.15789^{5}^
	CP	$\overset{ˆ}{a}$	0.872^{7.5}^	0.91^{3}^	0.894^{5}^	0.872^{7.5}^	0.938^{2}^	0.904^{4}^	0.89^{6}^	0.95^{1}^
		$\overset{ˆ}{b}$	0.88^{7}^	0.934^{2.5}^	0.896^{6}^	0.852^{8}^	0.934^{2.5}^	0.92^{4}^	0.914^{5}^	0.936^{1}^
	*D*_*abs*_		0.05806^{2}^	0.06004^{5}^	0.06197^{8}^	0.05483^{1}^	0.06192^{7}^	0.05842^{3}^	0.0609^{6}^	0.05974^{4}^
	*D*_*max*_		0.09366^{3}^	0.09547^{5}^	0.10192^{8}^	0.08649^{1}^	0.09858^{6}^	0.09093^{2}^	0.10105^{7}^	0.09546^{4}^
	*ASAE*		0.04597^{8}^	0.04189^{3}^	0.04261^{5}^	0.0434^{6}^	0.0425^{4}^	0.04175^{2}^	0.04367^{7}^	0.04051^{1}^
	∑*Ranks*		41.5^{2}^	48.5^{3}^	68^{6}^	40.5^{1}^	85.5^{8}^	51^{4}^	77^{7}^	56^{5}^

70	AESTs	$\overset{ˆ}{a}$	2.07992	2.0525	2.08702	1.88929	2.03281	1.93148	2.06854	2.05557
		$\overset{ˆ}{b}$	0.24855	0.25071	0.24961	0.25902	0.25294	0.25627	0.25096	0.25075
	BIAS	$\overset{ˆ}{a}$	0.25923^{1}^	0.2754^{2}^	0.35224^{8}^	0.28181^{3}^	0.33856^{6}^	0.29583^{4}^	0.3419^{7}^	0.29783^{5}^
		$\overset{ˆ}{b}$	0.01309^{1}^	0.01462^{2}^	0.01718^{7}^	0.01679^{6}^	0.01787^{8}^	0.01599^{5}^	0.01549^{3.5}^	0.01549^{3.5}^
	MSE	$\overset{ˆ}{a}$	0.11283^{1}^	0.13959^{4}^	0.22947^{8}^	0.11864^{2}^	0.20327^{6}^	0.13606^{3}^	0.22243^{7}^	0.15308^{5}^
		$\overset{ˆ}{b}$	0.00028^{1}^	0.00033^{2}^	0.00047^{7}^	0.00046^{6}^	5*e* − 04^{8}^	0.00042^{5}^	0.00038^{3.5}^	0.00038^{3.5}^
	MRE	$\overset{ˆ}{a}$	0.12962^{1}^	0.1377^{2}^	0.17612^{8}^	0.14091^{3}^	0.16928^{6}^	0.14792^{4}^	0.17095^{7}^	0.14892^{5}^
		$\overset{ˆ}{b}$	0.05234^{1}^	0.05848^{2}^	0.06871^{7}^	0.06714^{6}^	0.07146^{8}^	0.06395^{5}^	0.06197^{3.5}^	0.06197^{3.5}^
	CIL	$\overset{ˆ}{a}$	1.43212^{3}^	1.46868^{4}^	1.85873^{7}^	1.13385^{1}^	1.6608^{6}^	1.31396^{2}^	1.87523^{8}^	1.55481^{5}^
		$\overset{ˆ}{b}$	0.06596^{1}^	0.07223^{2}^	0.08^{7}^	0.07493^{4}^	0.08252^{8}^	0.07738^{5}^	0.0776^{6}^	0.07428^{3}^
	CP	$\overset{ˆ}{a}$	0.936^{3}^	0.946^{1}^	0.922^{5}^	0.84^{8}^	0.914^{6}^	0.902^{7}^	0.926^{4}^	0.94^{2}^
		$\overset{ˆ}{b}$	0.932^{3.5}^	0.948^{2}^	0.93^{5}^	0.858^{8}^	0.932^{3.5}^	0.91^{7}^	0.958^{1}^	0.926^{6}^
	*D*_*abs*_		0.02955^{2}^	0.03086^{5}^	0.03326^{8}^	0.02958^{3}^	0.03219^{7}^	0.03039^{4}^	0.03135^{6}^	0.02941^{1}^
	*D*_*max*_		0.04712^{1}^	0.04923^{5}^	0.05431^{8}^	0.04765^{3}^	0.05215^{7}^	0.04893^{4}^	0.0513^{6}^	0.04752^{2}^
	*ASAE*		0.02065^{8}^	0.01922^{2}^	0.01985^{6}^	0.01996^{7}^	0.01944^{4}^	0.0196^{5}^	0.01943^{3}^	0.01902^{1}^
	∑*Ranks*		32.5^{1}^	47^{3}^	89^{8}^	46^{2}^	82.5^{7}^	50^{5}^	73.5^{6}^	47.5^{4}^

150	AESTs	$\overset{ˆ}{a}$	2.03489	2.01795	2.04414	1.9244	2.01143	1.96307	2.04023	2.01985
		$\overset{ˆ}{b}$	0.24922	0.25052	0.25022	0.25453	0.25094	0.2538	0.25048	0.25023
	BIAS	$\overset{ˆ}{a}$	0.19476^{2}^	0.19845^{3}^	0.22953^{7}^	0.18435^{1}^	0.22039^{6}^	0.21434^{5}^	0.23129^{8}^	0.20026^{4}^
		$\overset{ˆ}{b}$	0.00959^{1}^	0.01049^{3}^	0.01175^{8}^	0.01022^{2}^	0.01146^{7}^	0.01111^{6}^	0.01062^{4}^	0.01084^{5}^
	MSE	$\overset{ˆ}{a}$	0.06324^{2}^	0.06472^{3}^	0.08965^{7}^	0.05002^{1}^	0.08122^{6}^	0.07016^{5}^	0.09067^{8}^	0.06623^{4}^
		$\overset{ˆ}{b}$	0.00014^{1}^	0.00017^{3}^	0.00022^{8}^	0.00016^{2}^	0.00021^{7}^	2*e* − 04^{6}^	0.00018^{4.5}^	0.00018^{4.5}^
	MRE	$\overset{ˆ}{a}$	0.09738^{2}^	0.09923^{3}^	0.11477^{7}^	0.09217^{1}^	0.11019^{6}^	0.10717^{5}^	0.11564^{8}^	0.10013^{4}^
		$\overset{ˆ}{b}$	0.03836^{1}^	0.04195^{3}^	0.04699^{8}^	0.0409^{2}^	0.04584^{7}^	0.04444^{6}^	0.04247^{4}^	0.04334^{5}^
	CIL	$\overset{ˆ}{a}$	0.9064^{2}^	0.95587^{4}^	1.13332^{7}^	0.79258^{1}^	1.07558^{6}^	0.91548^{3}^	1.17817^{8}^	0.96465^{5}^
		$\overset{ˆ}{b}$	0.04556^{1}^	0.04909^{3}^	0.05451^{7}^	0.04849^{2}^	0.05543^{8}^	0.05131^{5}^	0.05219^{6}^	0.04942^{4}^
	CP	$\overset{ˆ}{a}$	0.904^{6}^	0.926^{4.5}^	0.93^{2}^	0.87^{8}^	0.926^{4.5}^	0.898^{7}^	0.928^{3}^	0.936^{1}^
		$\overset{ˆ}{b}$	0.928^{5}^	0.938^{3}^	0.918^{6}^	0.902^{8}^	0.94^{2}^	0.904^{7}^	0.944^{1}^	0.936^{4}^
	*D*_*abs*_		0.01967^{1}^	0.02194^{6}^	0.02266^{8}^	0.02106^{3}^	0.02244^{7}^	0.02059^{2}^	0.02182^{5}^	0.02175^{4}^
	*D*_*max*_		0.03192^{1}^	0.03525^{5}^	0.03676^{8}^	0.03339^{2}^	0.03641^{7}^	0.03366^{3}^	0.03568^{6}^	0.035^{4}^
	*ASAE*		0.01266^{7}^	0.01222^{3}^	0.01262^{6}^	0.01291^{8}^	0.01232^{5}^	0.01218^{2}^	0.01231^{4}^	0.01196^{1}^
	∑*Ranks*		28^{2}^	49.5^{3}^	90^{8}^	27^{1}^	82.5^{7}^	54^{4}^	79.5^{6}^	57.5^{5}^

300	AESTs	$\overset{ˆ}{a}$	2.00902	2.01135	2.04042	1.96817	2.01772	1.9692	2.03231	2.00696
		$\overset{ˆ}{b}$	0.25046	0.25011	0.24919	0.25295	0.25004	0.25201	0.24986	0.25033
	BIAS	$\overset{ˆ}{a}$	0.13126^{1}^	0.1498^{5}^	0.16805^{7}^	0.13878^{2}^	0.16367^{6}^	0.14675^{4}^	0.17444^{8}^	0.13966^{3}^
		$\overset{ˆ}{b}$	0.00664^{1}^	0.0075^{3}^	0.00797^{5}^	0.00804^{6}^	0.00852^{8}^	0.00779^{4}^	0.00806^{7}^	0.00749^{2}^
	MSE	$\overset{ˆ}{a}$	0.02681^{1}^	0.03526^{5}^	0.04658^{7}^	0.03101^{2}^	0.04243^{6}^	0.03428^{4}^	0.05087^{8}^	0.03287^{3}^
		$\overset{ˆ}{b}$	7*e* − 05^{1}^	9*e* − 05^{3}^	1*e* − 04^{6}^	1*e* − 04^{6}^	0.00011^{8}^	9*e* − 05^{3}^	1*e* − 04^{6}^	9*e* − 05^{3}^
	MRE	$\overset{ˆ}{a}$	0.06563^{1}^	0.0749^{5}^	0.08403^{7}^	0.06939^{2}^	0.08184^{6}^	0.07337^{4}^	0.08722^{8}^	0.06983^{3}^
		$\overset{ˆ}{b}$	0.02655^{1}^	0.02999^{3}^	0.0319^{5}^	0.03216^{6}^	0.03408^{8}^	0.03117^{4}^	0.03222^{7}^	0.02994^{2}^
	CIL	$\overset{ˆ}{a}$	0.61913^{2}^	0.66049^{4}^	0.77102^{7}^	0.57935^{1}^	0.75332^{6}^	0.65221^{3}^	0.80491^{8}^	0.67189^{5}^
		$\overset{ˆ}{b}$	0.03225^{1}^	0.03459^{3}^	0.03826^{7}^	0.03327^{2}^	0.03887^{8}^	0.03548^{5}^	0.03679^{6}^	0.03496^{4}^
	CP	$\overset{ˆ}{a}$	0.928^{4}^	0.93^{2.5}^	0.922^{6}^	0.864^{8}^	0.932^{1}^	0.904^{7}^	0.924^{5}^	0.93^{2.5}^
		$\overset{ˆ}{b}$	0.946^{1}^	0.934^{3.5}^	0.932^{5}^	0.854^{8}^	0.922^{6}^	0.916^{7}^	0.934^{3.5}^	0.94^{2}^
	*D*_*abs*_		0.01433^{1}^	0.01478^{4}^	0.01598^{7}^	0.01471^{3}^	0.01531^{6}^	0.01469^{2}^	0.01666^{8}^	0.01519^{5}^
	*D*_*max*_		0.023^{1}^	0.0241^{4}^	0.02608^{7}^	0.02382^{2}^	0.02516^{6}^	0.02396^{3}^	0.0271^{8}^	0.0245^{5}^
	*ASAE*		0.00856^{8}^	0.00792^{1}^	0.00818^{3}^	0.00849^{7}^	0.00845^{6}^	0.00824^{4}^	0.00831^{5}^	0.00801^{2}^
	∑*Ranks*		36^{1}^	56^{5}^	72^{6}^	38^{2}^	78^{7}^	48^{3}^	85.5^{8}^	54.5^{4}^
500	AESTs	$\overset{ˆ}{a}$	2.01351	1.99957	2.00948	1.97657	2.0062	1.97824	2.01232	2.01253
		$\overset{ˆ}{b}$	0.24961	0.25032	0.25003	0.25168	0.25029	0.2514	0.24986	0.24983
	BIAS	$\overset{ˆ}{a}$	0.10232^{2}^	0.10917^{3}^	0.11996^{7}^	0.09913^{1}^	0.11884^{6}^	0.11199^{5}^	0.12973^{8}^	0.11014^{4}^
		$\overset{ˆ}{b}$	0.0055^{2}^	0.00587^{5}^	0.00624^{7}^	0.00542^{1}^	0.00618^{6}^	0.00573^{4}^	0.00656^{8}^	0.00558^{3}^
	MSE	$\overset{ˆ}{a}$	0.01652^{2}^	0.01912^{3}^	0.02284^{6}^	0.01643^{1}^	0.02304^{7}^	0.01953^{4}^	0.02748^{8}^	0.02028^{5}^
		$\overset{ˆ}{b}$	5*e* − 05^{3}^	5*e* − 05^{3}^	6*e* − 05^{7}^	5*e* − 05^{3}^	6*e* − 05^{7}^	5*e* − 05^{3}^	6*e* − 05^{7}^	5*e* − 05^{3}^
	MRE	$\overset{ˆ}{a}$	0.05116^{2}^	0.05458^{3}^	0.05998^{7}^	0.04956^{1}^	0.05942^{6}^	0.056^{5}^	0.06486^{8}^	0.05507^{4}^
		$\overset{ˆ}{b}$	0.022^{2}^	0.0235^{5}^	0.02496^{7}^	0.02167^{1}^	0.02472^{6}^	0.0229^{4}^	0.02624^{8}^	0.0223^{3}^
	CIL	$\overset{ˆ}{a}$	0.47929^{2}^	0.50153^{3}^	0.57934^{7}^	0.45251^{1}^	0.57397^{6}^	0.51002^{4}^	0.60989^{8}^	0.5115^{5}^
		$\overset{ˆ}{b}$	0.02497^{1}^	0.02658^{3}^	0.02982^{7}^	0.02548^{2}^	0.02998^{8}^	0.02725^{5}^	0.02852^{6}^	0.02678^{4}^
	CP	$\overset{ˆ}{a}$	0.936^{2}^	0.934^{3}^	0.938^{1}^	0.9^{8}^	0.93^{4}^	0.926^{5.5}^	0.924^{7}^	0.926^{5.5}^
		$\overset{ˆ}{b}$	0.922^{6}^	0.928^{5}^	0.936^{2.5}^	0.896^{8}^	0.932^{4}^	0.92^{7}^	0.936^{2.5}^	0.938^{1}^
	*D*_*abs*_		0.01145^{2}^	0.01168^{4}^	0.01191^{5}^	0.01138^{1}^	0.01212^{7}^	0.01157^{3}^	0.01241^{8}^	0.01207^{6}^
	*D*_*max*_		0.01825^{2}^	0.01887^{4}^	0.01937^{6}^	0.01821^{1}^	0.01965^{7}^	0.01869^{3}^	0.02035^{8}^	0.01929^{5}^
	*ASAE*		0.00625^{8}^	0.00593^{1}^	0.00599^{3}^	0.00623^{7}^	0.00604^{4.5}^	0.00608^{6}^	0.00604^{4.5}^	0.00596^{2}^
	∑*Ranks*		38^{2}^	47^{3}^	83.5^{7}^	22^{1}^	80.5^{6}^	51.5^{4}^	90^{8}^	55.5^{5}^

**Table 5:** Simulation values of the WDR model for (*a* = 4, *b* = 3).

n	Est.	Est. Par.	MLEs	ADEs	CVMEs	MPSEs	OLSEs	PCEs	RTADEs	WLSEs
20	AESTs	$\overset{ˆ}{a}$	4.98939	4.42485	5.82656	3.65696	4.41757	3.88811	5.84177	4.84927
		$\overset{ˆ}{b}$	2.96003	3.04813	2.93143	3.22962	3.1034	3.17503	2.95778	3.07964
	BIAS	$\overset{ˆ}{a}$	1.65347^{4}^	1.41568^{3}^	2.66031^{7}^	1.33029^{1}^	1.75682^{5}^	1.34785^{2}^	2.78628^{8}^	2.13146^{6}^
		$\overset{ˆ}{b}$	0.27945^{1}^	0.29616^{2}^	0.34615^{6}^	0.37006^{7}^	0.3743^{8}^	0.34602^{5}^	0.33899^{3}^	0.34398^{4}^
	MSE	$\overset{ˆ}{a}$	9.83351^{4}^	5.70959^{3}^	43.99055^{6}^	3.75162^{2}^	14.14101^{5}^	3.16739^{1}^	46.78042^{7}^	130.11327^{8}^
		$\overset{ˆ}{b}$	0.12267^{1}^	0.13446^{2}^	0.18052^{4}^	0.21697^{7}^	0.22678^{8}^	0.19493^{6}^	0.17339^{3}^	0.18849^{5}^
	MRE	$\overset{ˆ}{a}$	0.41337^{4}^	0.35392^{3}^	0.66508^{7}^	0.33257^{1}^	0.4392^{5}^	0.33696^{2}^	0.69657^{8}^	0.53287^{6}^
		$\overset{ˆ}{b}$	0.09315^{1}^	0.09872^{2}^	0.11538^{6}^	0.12335^{7}^	0.12477^{8}^	0.11534^{5}^	0.113^{3}^	0.11466^{4}^
	CIL	$\overset{ˆ}{a}$	13.34119^{4}^	9.95014^{3}^	32.94815^{7}^	5.47103^{1}^	13.36871^{5}^	6.67832^{2}^	33.28162^{8}^	14.7216^{6}^
		$\overset{ˆ}{b}$	1.29155^{1}^	1.47339^{2}^	1.56058^{4}^	1.68812^{6}^	1.78715^{8}^	1.7083^{7}^	1.55807^{3}^	1.65292^{5}^
	CP	$\overset{ˆ}{a}$	0.886^{6}^	0.944^{2}^	0.878^{7}^	0.812^{8}^	0.948^{1}^	0.888^{4.5}^	0.888^{4.5}^	0.926^{3}^
		$\overset{ˆ}{b}$	0.886^{6}^	0.95^{1}^	0.878^{7}^	0.842^{8}^	0.93^{3}^	0.89^{5}^	0.908^{4}^	0.932^{2}^
	*D*_*abs*_		0.05616^{1}^	0.05736^{2}^	0.05978^{5}^	0.05807^{4}^	0.06109^{7}^	0.05772^{3}^	0.06375^{8}^	0.06025^{6}^
	*D*_*max*_		0.09126^{3}^	0.09122^{2}^	0.09965^{7}^	0.09172^{4}^	0.09762^{6}^	0.09107^{1}^	0.10572^{8}^	0.09732^{5}^
	*ASAE*		0.04625^{8}^	0.0419^{3}^	0.04324^{5}^	0.04374^{7}^	0.0417^{2}^	0.04244^{4}^	0.04336^{6}^	0.04116^{1}^
	∑*Ranks*		39^{1}^	51^{2}^	64^{6}^	56^{4}^	75^{8}^	53.5^{3}^	70.5^{7}^	59^{5}^

70	AESTs	$\overset{ˆ}{a}$	4.20899	4.18661	4.31187	3.68368	4.13213	3.82434	4.31306	4.10577
		$\overset{ˆ}{b}$	2.98603	2.99953	2.97448	3.10557	3.01998	3.07492	2.99268	3.00984
	BIAS	$\overset{ˆ}{a}$	0.68241^{2}^	0.73054^{5}^	0.85888^{7}^	0.63551^{1}^	0.80574^{6}^	0.71155^{4}^	0.90453^{8}^	0.70668^{3}^
		$\overset{ˆ}{b}$	0.15406^{1}^	0.16018^{2}^	0.17855^{7}^	0.17227^{5}^	0.17879^{8}^	0.17425^{6}^	0.171^{4}^	0.1602^{3}^
	MSE	$\overset{ˆ}{a}$	0.83208^{3}^	0.97591^{5}^	1.37516^{7}^	0.60866^{1}^	1.22401^{6}^	0.77034^{2}^	1.51198^{8}^	0.91749^{4}^
		$\overset{ˆ}{b}$	0.03615^{1}^	0.03933^{3}^	0.04912^{6}^	0.04644^{5}^	0.0504^{8}^	0.04987^{7}^	0.04546^{4}^	0.03857^{2}^
	MRE	$\overset{ˆ}{a}$	0.1706^{2}^	0.18263^{5}^	0.21472^{7}^	0.15888^{1}^	0.20144^{6}^	0.17789^{4}^	0.22613^{8}^	0.17667^{3}^
		$\overset{ˆ}{b}$	0.05135^{1}^	0.05339^{2}^	0.05952^{7}^	0.05742^{5}^	0.0596^{8}^	0.05808^{6}^	0.057^{4}^	0.0534^{3}^
	CIL	$\overset{ˆ}{a}$	3.56176^{3}^	3.71685^{4}^	4.71912^{7}^	2.57667^{1}^	4.17857^{6}^	3.0767^{2}^	4.98854^{8}^	3.77883^{5}^
		$\overset{ˆ}{b}$	0.69754^{1}^	0.75665^{2}^	0.83045^{6}^	0.78459^{4}^	0.86642^{8}^	0.82052^{5}^	0.83216^{7}^	0.77978^{3}^
	CP	$\overset{ˆ}{a}$	0.908^{6}^	0.944^{1}^	0.928^{4}^	0.824^{8}^	0.932^{3}^	0.872^{7}^	0.922^{5}^	0.94^{2}^
		$\overset{ˆ}{b}$	0.914^{6}^	0.94^{2}^	0.922^{5}^	0.856^{8}^	0.932^{3}^	0.896^{7}^	0.924^{4}^	0.942^{1}^
	*D*_*abs*_		0.03^{1}^	0.03012^{3}^	0.03271^{8}^	0.03004^{2}^	0.03207^{6}^	0.03055^{4}^	0.03236^{7}^	0.03076^{5}^
	*D*_*max*_		0.04809^{1}^	0.04872^{3}^	0.05363^{7}^	0.04815^{2}^	0.0522^{6}^	0.04917^{4}^	0.05372^{8}^	0.04969^{5}^
	*ASAE*		0.01975^{6}^	0.01897^{2}^	0.02008^{7}^	0.02027^{8}^	0.01939^{3}^	0.01965^{4}^	0.01967^{5}^	0.01895^{1}^
	∑*Ranks*		28^{1}^	51^{3}^	85^{8}^	37^{2}^	83^{7}^	52^{4.5}^	80^{6}^	52^{4.5}^

150	AESTs	$\overset{ˆ}{a}$	4.09098	4.03974	4.16026	3.88263	4.03204	3.91074	4.17111	4.07102
		$\overset{ˆ}{b}$	2.99467	3.00916	2.98883	3.04614	3.00953	3.034	2.98974	3.00644
	BIAS	$\overset{ˆ}{a}$	0.43466^{3}^	0.46102^{4}^	0.57865^{7}^	0.41404^{1}^	0.51526^{6}^	0.42871^{2}^	0.62673^{8}^	0.46332^{5}^
		$\overset{ˆ}{b}$	0.10507^{1}^	0.10777^{2}^	0.12735^{7}^	0.10979^{4}^	0.11861^{6}^	0.10898^{3}^	0.12886^{8}^	0.11105^{5}^
	MSE	$\overset{ˆ}{a}$	0.32016^{3}^	0.34641^{4}^	0.54603^{7}^	0.27672^{1}^	0.45772^{6}^	0.28554^{2}^	0.65691^{8}^	0.3858^{5}^
		$\overset{ˆ}{b}$	0.01675^{1}^	0.01753^{2}^	0.02462^{7}^	0.0187^{4}^	0.02223^{6}^	0.01916^{5}^	0.02497^{8}^	0.01867^{3}^
	MRE	$\overset{ˆ}{a}$	0.10867^{3}^	0.11525^{4}^	0.14466^{7}^	0.10351^{1}^	0.12881^{6}^	0.10718^{2}^	0.15668^{8}^	0.11583^{5}^
		$\overset{ˆ}{b}$	0.03502^{1}^	0.03592^{2}^	0.04245^{7}^	0.0366^{4}^	0.03954^{6}^	0.03633^{3}^	0.04295^{8}^	0.03702^{5}^
	CIL	$\overset{ˆ}{a}$	2.1919^{3}^	2.28521^{4}^	2.81395^{7}^	1.87985^{1}^	2.61523^{6}^	2.11242^{2}^	3.00267^{8}^	2.35732^{5}^
		$\overset{ˆ}{b}$	0.47968^{1}^	0.51679^{3}^	0.5703^{7}^	0.50669^{2}^	0.58058^{8}^	0.53828^{5}^	0.56398^{6}^	0.51916^{4}^
	CP	$\overset{ˆ}{a}$	0.93^{5}^	0.938^{1}^	0.936^{3}^	0.888^{8}^	0.936^{3}^	0.908^{7}^	0.912^{6}^	0.936^{3}^
		$\overset{ˆ}{b}$	0.924^{4}^	0.952^{2}^	0.922^{5}^	0.886^{8}^	0.942^{3}^	0.92^{6}^	0.914^{7}^	0.954^{1}^
	*D*_*abs*_		0.02008^{1}^	0.02141^{6}^	0.02178^{7}^	0.02054^{3}^	0.02025^{2}^	0.02091^{5}^	0.02213^{8}^	0.02069^{4}^
	*D*_*max*_		0.03236^{1}^	0.03453^{6}^	0.036^{7}^	0.03268^{2}^	0.03325^{3}^	0.03347^{5}^	0.03673^{8}^	0.03326^{4}^
	*ASAE*		0.01271^{8}^	0.01203^{1}^	0.01242^{6}^	0.01223^{3}^	0.01253^{7}^	0.01226^{4}^	0.01228^{5}^	0.01216^{2}^
	∑*Ranks*		35^{2}^	53^{4}^	86^{7}^	28^{1}^	74^{6}^	43^{3}^	88^{8}^	61^{5}^

300	AESTs	$\overset{ˆ}{a}$	4.04824	4.00475	4.0426	3.93561	4.00653	3.89932	4.03998	4.03372
		$\overset{ˆ}{b}$	2.9956	3.00247	2.99965	3.02136	3.00738	3.02832	3.00223	3.00071
	BIAS	$\overset{ˆ}{a}$	0.312^{3}^	0.30434^{2}^	0.35513^{6}^	0.27491^{1}^	0.36662^{7}^	0.34466^{5}^	0.41168^{8}^	0.33723^{4}^
		$\overset{ˆ}{b}$	0.07292^{3}^	0.0726^{2}^	0.08126^{5}^	0.07136^{1}^	0.0877^{7}^	0.08415^{6}^	0.09091^{8}^	0.0793^{4}^
	MSE	$\overset{ˆ}{a}$	0.15597^{3}^	0.15435^{2}^	0.21079^{6}^	0.13167^{1}^	0.21737^{7}^	0.17787^{5}^	0.28187^{8}^	0.17364^{4}^
		$\overset{ˆ}{b}$	0.00818^{2}^	0.00825^{3}^	0.01077^{5}^	0.00783^{1}^	0.01191^{7}^	0.01108^{6}^	0.0129^{8}^	0.00936^{4}^
	MRE	$\overset{ˆ}{a}$	0.078^{3}^	0.07608^{2}^	0.08878^{6}^	0.06873^{1}^	0.09166^{7}^	0.08616^{5}^	0.10292^{8}^	0.08431^{4}^
		$\overset{ˆ}{b}$	0.02431^{3}^	0.0242^{2}^	0.02709^{5}^	0.02379^{1}^	0.02923^{7}^	0.02805^{6}^	0.0303^{8}^	0.02643^{4}^
	CIL	$\overset{ˆ}{a}$	1.47503^{2}^	1.58008^{4}^	1.85191^{7}^	1.35934^{1}^	1.80934^{6}^	1.49414^{3}^	1.92968^{8}^	1.60974^{5}^
		$\overset{ˆ}{b}$	0.33825^{1}^	0.36535^{3}^	0.40724^{7}^	0.34805^{2}^	0.41021^{8}^	0.37443^{5}^	0.3981^{6}^	0.36536^{4}^
	CP	$\overset{ˆ}{a}$	0.934^{3}^	0.942^{1}^	0.932^{4}^	0.916^{6}^	0.93^{5}^	0.884^{8}^	0.912^{7}^	0.936^{2}^
		$\overset{ˆ}{b}$	0.93^{5}^	0.946^{1}^	0.932^{3.5}^	0.924^{6}^	0.932^{3.5}^	0.892^{8}^	0.912^{7}^	0.94^{2}^
	*D*_*abs*_		0.01457^{3}^	0.01442^{2}^	0.01497^{5}^	0.01365^{1}^	0.0153^{7}^	0.0146^{4}^	0.01567^{8}^	0.01516^{6}^
	*D*_*max*_		0.02346^{3}^	0.0231^{2}^	0.0244^{5}^	0.02182^{1}^	0.02487^{7}^	0.02378^{4}^	0.02595^{8}^	0.02449^{6}^
	*ASAE*		0.0083^{8}^	0.00794^{2}^	0.00826^{5}^	0.00827^{6}^	0.00815^{4}^	0.00828^{7}^	0.00808^{3}^	0.00786^{1}^
	∑*Ranks*		44^{3}^	42^{2}^	72.5^{6}^	23^{1}^	83.5^{7}^	58^{4}^	85^{8}^	60^{5}^
500	AESTs	$\overset{ˆ}{a}$	4.04932	3.99242	4.04301	3.93307	4.00964	3.93894	4.03144	3.99752
		$\overset{ˆ}{b}$	2.993	3.00154	2.9971	3.01842	3.00586	3.01781	3.00206	3.00547
	BIAS	$\overset{ˆ}{a}$	0.23155^{2}^	0.24762^{4}^	0.28474^{6}^	0.2173^{1}^	0.29061^{7}^	0.24685^{3}^	0.31634^{8}^	0.25335^{5}^
		$\overset{ˆ}{b}$	0.05377^{1}^	0.05958^{3}^	0.06738^{8}^	0.05916^{2}^	0.067^{7}^	0.05983^{4}^	0.06539^{6}^	0.06243^{5}^
	MSE	$\overset{ˆ}{a}$	0.08442^{1}^	0.09899^{3}^	0.13148^{6}^	0.08575^{2}^	0.13333^{7}^	0.09969^{4}^	0.15584^{8}^	0.10381^{5}^
		$\overset{ˆ}{b}$	0.00446^{1}^	0.00574^{3}^	0.0073^{8}^	0.00542^{2}^	0.00704^{7}^	0.00587^{4}^	0.00651^{6}^	0.00604^{5}^
	MRE	$\overset{ˆ}{a}$	0.05789^{2}^	0.0619^{4}^	0.07118^{6}^	0.05433^{1}^	0.07265^{7}^	0.06171^{3}^	0.07908^{8}^	0.06334^{5}^
		$\overset{ˆ}{b}$	0.01792^{1}^	0.01986^{3}^	0.02246^{8}^	0.01972^{2}^	0.02233^{7}^	0.01994^{4}^	0.0218^{6}^	0.02081^{5}^
	CIL	$\overset{ˆ}{a}$	1.13431^{2}^	1.20529^{4}^	1.40542^{7}^	1.05703^{1}^	1.37279^{6}^	1.17453^{3}^	1.48708^{8}^	1.21354^{5}^
		$\overset{ˆ}{b}$	0.26213^{1}^	0.28161^{3}^	0.31397^{7}^	0.26818^{2}^	0.3157^{8}^	0.28677^{5}^	0.30975^{6}^	0.28319^{4}^
	CP	$\overset{ˆ}{a}$	0.934^{4}^	0.942^{1}^	0.914^{6}^	0.91^{7}^	0.938^{2}^	0.906^{8}^	0.932^{5}^	0.936^{3}^
		$\overset{ˆ}{b}$	0.93^{3}^	0.928^{4}^	0.914^{6}^	0.906^{7.5}^	0.932^{2}^	0.906^{7.5}^	0.936^{1}^	0.926^{5}^
	*D*_*abs*_		0.01085^{1}^	0.01097^{2}^	0.0123^{8}^	0.01128^{3}^	0.01207^{6}^	0.01135^{4}^	0.01214^{7}^	0.01202^{5}^
	*D*_*max*_		0.01748^{1}^	0.01778^{2}^	0.02001^{7}^	0.01795^{3}^	0.01969^{6}^	0.01829^{4}^	0.0201^{8}^	0.01932^{5}^
	*ASAE*		0.00602^{6}^	0.00576^{1}^	0.006^{4.5}^	0.00605^{8}^	0.006^{4.5}^	0.00603^{7}^	0.00599^{3}^	0.00587^{2}^
	∑*Ranks*		30^{1}^	45^{3}^	81.5^{6}^	30.5^{2}^	86.5^{8}^	47.5^{4}^	86^{7}^	61^{5}^

**Table 6:** Partial and overall ranks of all the methods of estimation of WDR distribution by various values of *a* and *b*.

Parameter	*n*	MLEs	ADEs	CVMEs	MPSEs	OLSEs	PCEs	RTADEs	WLSEs
*a* = 0.25, *b* = 0.75	20	1	3	6	2	7	8	4	5
	70	1	3	6	2	7	8	5	4
	150	2	3	6	1	8	7	5	4
	300	5	2	7	1	8	6	4	3
	500	5	2	8	1	6	7	4	3
*a* = 0.5, *b* = 2	20	1	2	6	3	8	7	4	5
	70	1	3	6	2	8	7	5	4
	150	1	4	7	2	8	6	5	3
	300	1	3.5	6	2	8	7	5	3.5
	500	1	4	6	2	7	8	5	3
*a* = 3, *b* = 1.5	20	2	1	7	4	8	3	5	6
	70	1	4	8	2	6	3	7	5
	150	2	3	8	1	6	5	7	4
	300	2	3	8	1	7	5	6	4
	500	2	5	8	1	7	4	6	3
*a* = 2, *b* = 0.25	20	2	3	6	1	8	4	7	5
	70	1	3	8	2	7	5	6	4
	150	2	3	8	1	7	4	6	5
	300	1	5	6	2	7	3	8	4
	500	2	3	7	1	6	4	8	5
*a* = 4, *b* = 3	20	1	2	6	4	8	3	7	5
	70	1	3	8	2	7	4.5	6	4.5
	150	2	4	7	1	6	3	8	5
	300	3	2	6	1	7	4	8	5
	500	1	3	6	2	8	4	7	5

∑ Ranks		44.0	76.5	171.0	44.0	180.0	129.5	148.0	107.0
Overall Rank		1.5	3	7	1.5	8	5	6	4

#### 4.2.1 Concluding remarks on simulation results

We cannot compare the performance of the different estimators theoretically. For this reason, we conduct a simulation study by generating random samples from the proposed distribution and use the ABBs, MSEs, MREs, $D_{abs}$, $D_{max}$ and ASAE criteria to compare the performance of the different estimators. The simulation results show the following remarks:

• All different estimation methods estimates have the consistency property that leads to their initial values as the sample size increases.
• All criteria used in our comparison decrease as the sample size increases, except the CP increases.
• From Table 1, Table 2, Table 3, Table 4, Table 5, we can say that all estimation methods behave very well for the estimation proposed model parameters.
• The simulation results show that the MLEs and MPSEs perform better than the other estimation methods in most cases, as shown in Table 6. So, we can recommend using both of them to estimate the unknown parameters when we ensure that the data come from the proposed distribution.

## 5 Actuarial measures

One of the primary tasks of actuarial science institutions is to predict the market risks in a portfolio of instruments. Henceforth, estimating the risk measures is very important in selling and buying products. Risk measurements are investigated for the WDR distribution in this part. Five risk measures are explored for the WDR distribution. Some actuarial measures such as VaR, TVaR, TV, TVP, and ES are calculated. A brief simulation study for these measures is provided. Finally, an application to the insurance loss data set is analyzed. The computing of these actuarial measures allows organizations to gain a comprehensive view of their risk landscape, enabling them to manage risks more effectively, comply with regulatory requirements, and make informed strategic decisions to ensure financial stability and growth.

### 5.1 VaR measure

The VaR, also known as the quantile risk measure or the idea of quantile premiums, is often specified with a predetermined level of confidence, denoted by the symbol q (commonly 90%, 95%, or 99% of the total value). This level of confidence is used when calculating the VaR. In contrast, VaR refers to a quantitative total of the cumulative loss distribution (see [10]).

The WDR distribution's VaR value is calculated as follows:

$$VaR_{q}=b\sqrt{\log\left\{\frac{1}{{\left[1-{\left(2^{p}-1\right)}^{1/a}\right]}^{2}}\right\}}.$$

### 5.2 TVaR measure

Among the actuarial measures, TVaR is the most prominent one and deals with the tail variance beyond VaR. It estimates the worth of a prospective loss when an event occurs outside of the predetermined probability. It is defined as follows

$$TVaR_{q}=\frac{1}{\left(1-q\right)}\int_{VaR_{q}}^{\infty }x\;\;f(x)dx.$$

The WDR distribution has the following TVaR value:

(6) $$TVaR_{q}=\frac{1}{1-q}\sum_{k=1}^{\infty }\sum_{m=0}^{\infty }\Psi_{k,m}\int_{VaR_{q}}^{\infty }xh_{m}(x)=\frac{1}{1-q}\sum_{k=1}^{\infty }\sum_{m=0}^{\infty }\Psi_{k,m}\left[Ve^{-\frac{mV^{2}}{2b^{2}}}+\frac{\sqrt{\frac{\pi }{2}}b\;\text{erfc}\left(\frac{\sqrt{m}V}{\sqrt{2}b}\right)}{\sqrt{m}}\right],$$

where *V* is $VaR_{q}$ and $\text{erfc}(z)=1-\frac{2}{\sqrt{\pi }}\int_{0}^{z}e^{-t^{2}}dt$.

### 5.3 TVP measure

In this subsection, we discuss another most important risk measure called TVP. Landsman [25] revealed the TV risk, which is described as the deviation of the loss distribution that is bigger than a critical value. It is said that the WDR distribution's TV is

(7) $$TV_{q}(X)=E(X^{2}|X>x_{q})-{\left(TVaR_{q}\right)}^{2}=\frac{1}{\left(1-q\right)}\int_{VaR_{q}}^{\infty }x^{2}f(x)dx-{\left(TVaR_{q}\right)}^{2},$$

where

(8) $$E(X^{2}|X>x_{q})=\frac{1}{1-q}\sum_{k=1}^{\infty }\sum_{m=0}^{\infty }\Psi_{k,m}\frac{e^{-\frac{mV^{2}}{2b^{2}}}\left(2b^{2}+mV^{2}\right)}{m},$$

by using Equation (6)–(8), we get the TV of the WDR distribution.

In the insurance industry, the TVP is also a useful criterion. The WDR distribution's TVP can be written below

(9) $$TVP_{q}(x)=TVaR_{q}+q\;TV_{q},$$

where 0<q<1. The TVP of the WDR distribution follows by replacing Equations (6) and (7) in Equation (9).

### 5.4 ES measure

The ES measure is used in financial risk measurement to evaluate a portfolio's market or credit risk. ES estimates the risk of an investment in a conservative way, focusing on the less profitable outcomes. It is considered a more useful risk measure than VaR because it is a coherent spectral measure of financial portfolio risk. Financial risk analysts have established the expected shortfall ES as a standard indicator. Definition of WDR's ES in the following form

(10) $$ES_{q}(x)=\frac{1}{q}\int_{0}^{q}VaR_{t}\;dt=\frac{b}{q}\int_{0}^{q}\sqrt{\log\left\{\frac{1}{{\left[1-{\left(2^{t}-1\right)}^{1/a}\right]}^{2}}\right\}}\;dt.$$

### 5.5 Numerical simulations for risk measures

**Figure 3:**
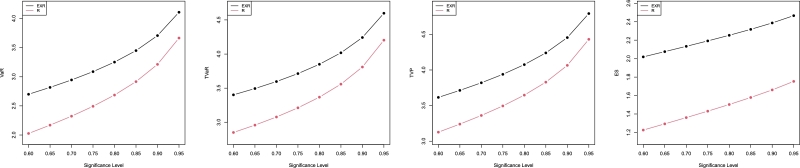
Plots of risk measures using the values in Table 7.

**Figure 4:**
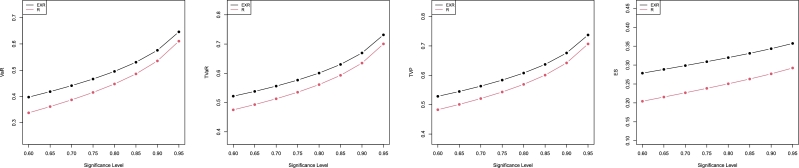
Plots of risk measures using the values in Table 8.

Some empirical findings for the risk mentioned above metrics, WDR and R distributions, are discussed in this section. Table 7, Table 8 offer simulation results for the “WDR” and “R” distributions' average VaR, TVaR, TVP, and ES, and displayed graphically in Figure 3, Figure 4.

**Table 7:** Simulation outcomes for the four risk measures for the WDR and RD.

Model	Par.	*q* value	VaR	TVaR	TVP	ES
WDR	*a* = 3, *b* = 1.5	0.60	2.69895	3.40241	3.61508	2.01916
		0.65	2.81684	3.49458	3.71338	2.07593
		0.70	2.94481	3.59704	3.82007	2.13335
		0.75	3.08717	3.71353	3.93865	2.19210
		0.80	3.25081	3.85021	4.07481	2.25302
		0.85	3.44814	4.01831	4.23902	2.31731
		0.90	3.70602	4.24241	4.45422	2.38691
		0.95	4.10633	4.59792	4.79133	2.46586

R	*b* = 1.5	0.60	2.02630	2.85091	3.12713	1.22605
		0.65	2.16893	2.95863	3.23996	1.29304
		0.70	2.32271	3.07762	3.36131	1.36102
		0.75	2.49238	3.21195	3.49493	1.43068
		0.80	2.68549	3.36829	3.64696	1.50292
		0.85	2.91564	3.55885	3.82860	1.57902
		0.90	3.21214	3.81013	4.06436	1.66109
		0.95	3.66386	4.20321	4.42971	1.75351

**Table 8:** Simulation outcomes for the four risk measures for the WDR and RD.

Model	Par.	*q* value	VaR	TVaR	TVP	ES
WDR	*a* = 2, *b* = 0.25	0.60	0.39755	0.52188	0.52844	0.27862
		0.65	0.41848	0.53816	0.54490	0.28857
		0.70	0.44120	0.55624	0.56310	0.29865
		0.75	0.46646	0.57678	0.58367	0.30898
		0.80	0.49546	0.60083	0.60768	0.31970
		0.85	0.53037	0.63034	0.63704	0.33103
		0.90	0.57585	0.66956	0.67595	0.34330
		0.95	0.64609	0.73149	0.73727	0.35721

R	*b* = 0.25	0.60	0.33771	0.47514	0.48282	0.20434
		0.65	0.36148	0.49310	0.50091	0.21550
		0.70	0.38711	0.51293	0.52081	0.22683
		0.75	0.41539	0.53531	0.54318	0.23844
		0.80	0.44757	0.56137	0.56911	0.25048
		0.85	0.48593	0.59313	0.60062	0.26317
		0.90	0.53535	0.63501	0.64207	0.27684
		0.95	0.61063	0.70052	0.70681	0.29225

By analyzing the numerical values in Table 7, Table 8 of the calculated actuarial measures, we can conclude that the proposed model has the highest values for all calculated measures. Also, these measures increase by increasing the *q* value. Then, we conclude that the proposed model has a heavier tail than the R distribution and could be used efficiently to model heavy-tailed real data sets.

## 6 Application

**Figure 5:**
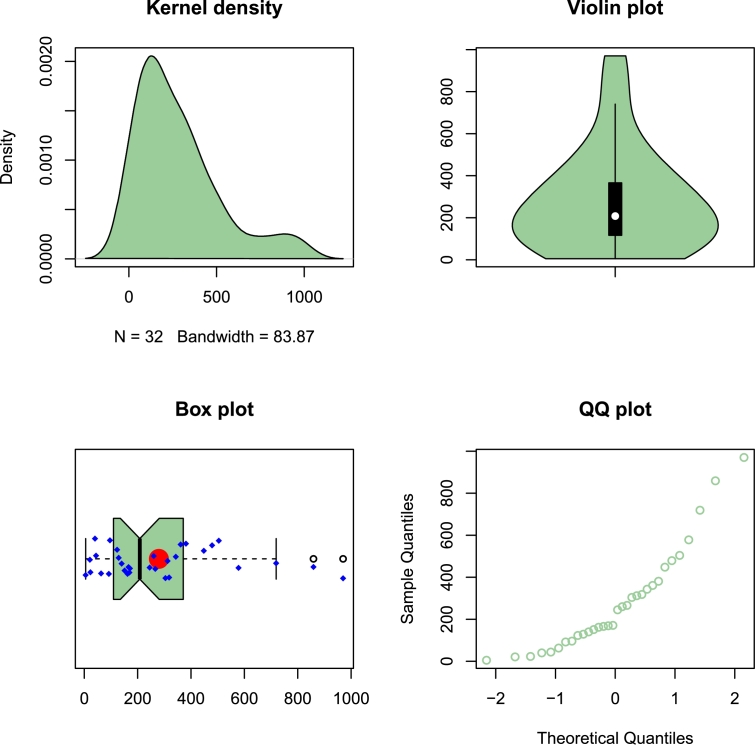
Non-parametric plots of the insurance real data set.

Here, we use an insurance data set to demonstrate the distribution's adaptability. The real data set presents the private passenger car insurance claims in the UK. It consists of four variables, and each variable has 32 observations. We focused on the variable number four (the number of claims). On the R software library, you may find these data sets; for more information about the analyzed real data set, see [28]. The numerical values of our real data set are 21, 40, 23, 5, 63, 171, 92, 44, 140, 343, 318, 129, 123, 448, 361, 169, 151, 479, 381, 166, 245, 970, 719, 304, 266, 859, 504, 162, 260, 578, 312, 96 and their descriptive statistics are introduced in Table 9. Some non-parametric plots of the analyzed real data sets are presented in Fig. 5.

**Table 9:** Descriptive statistics for the insurance real data set.

n	Min	First Quartile	Median	Mean	Third Quartile	Max	Skewness	Kurtosis
32	5.0	116.2	208.0	279.4	366.0	970.0	1.253	1.25313

The WDR distribution is evaluated in comparison to many other distributions, such as the area-biased Rayleigh distribution (ABR) [14], exponentiated inverse Rayleigh (ExIR) [37], exponentiated Rayleigh (ExR) [45], generalized Rayleigh (GR) [47], half logistic Inverse Rayleigh (HLIR) [6], inverse Rayleigh (IR) [2], modified Inverse Rayleigh (MIR) [24], Odd Lindley Rayleigh (OLR) [23], Rayleigh (R), transmuted Inverse Rayleigh (TIR) [2], transmuted Rayleigh (TR) [26], two-parameter Rayleigh (TPR) [18] and Weibull Raylieh (WR) [27] distributions.

**Figure 6:**
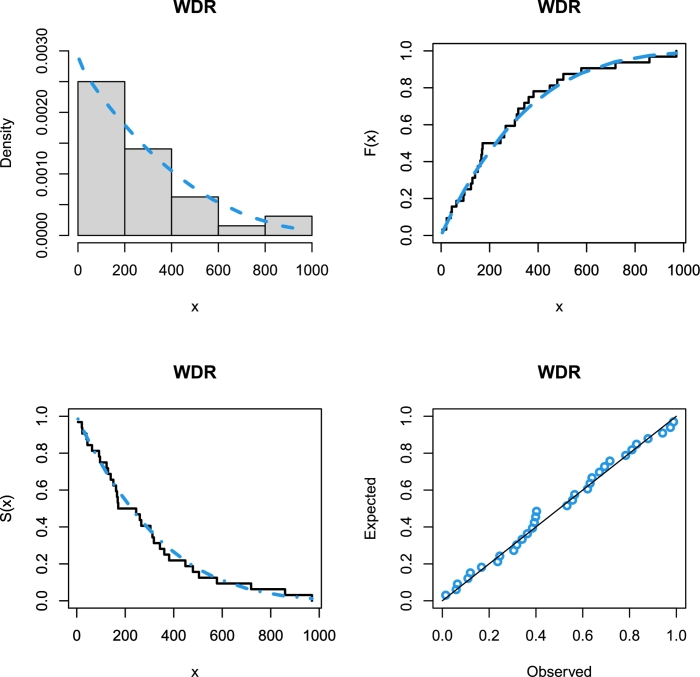
Histogram of the real data set with the fitted WDR model PDF, CDF, SF, and P-P plots.

The competing models were compared using some analytical measures including some information criteria (IC) such as Akaike IC (AIC), corrected AIC (CAIC), Bayesian IC (BIC), and Hannan–Quinn IC (HQIC) along with some goodness of fit measures such as Anderson Darling (AD), Cramér–von Mises (CM) and Kolmogorov–Smirnov (KS) with its *p*-value (KS *p*-value) to determine the best fitting model for the considered data set. The MLE approach has been chosen to estimate the parameters of the alternative models, and the analytical measures have been calculated using the help of the Wolfram Mathematica program, version 12.0. Table 10 offers the analytical measures in addition to the ML estimates and includes the standard errors (SEs) of those estimates in parentheses. Fig. 6 represents the WDR model's fitted PDF, CDF, SF, and P-P plots for the actual data set. These plots were generated using the data. The results in Table 10 indicate that the WDR distribution provides better fits than other competing models for the considered data set.

**Table 10:** The analytical measures and MLEs of the WDR model and other competing models for the considered real data set.

Model	*L*	AIC	CAIC	BIC	HQIC	AD	CM	KS	KS *p*-value	Est. parameters (SEs)
WDR	-211.804	427.608	428.022	430.539	428.58	0.166395	0.0265833	0.0973316	0.922223	$\overset{ˆ}{a}=0.492454\;(0.0934997)$
										$\overset{ˆ}{b}=372.29\;(59.3013)$

ABR	-257.718	517.435	517.569	518.901	517.921	19.2955	1.5191	0.429001	0.000015	$\overset{ˆ}{\sigma }=183.464\;(11.4665)$

ExIR	-233.576	471.152	471.566	474.084	472.124	5.31187	1.07879	0.339663	0.001242	$\overset{ˆ}{a}=0.186727\;(0.0358214)$
										$\overset{ˆ}{b}=12.4378\;(2.59959)$

ExR	-212.036	428.072	428.486	431.004	429.044	0.224223	0.0374367	0.111084	0.82462	$\overset{ˆ}{a}=0.428858\;(0.0880248)$
										$\overset{ˆ}{b}=353.236\;(53.2778)$

GR	-211.963	427.925	428.339	430.857	428.897	0.196569	0.0320802	0.107842	0.850711	$\overset{ˆ}{\theta }=3.2445\times 10^{-6}\;(1.09108\times 10^{-6})$
										$\overset{ˆ}{\alpha }=-0.563175\;(0.0890416)$

HLIR	-229.113	462.226	462.64	465.158	463.198	4.5621	0.926078	0.318386	0.003044	$\overset{ˆ}{\alpha }=11.9464\;(2.5797)$
										$\overset{ˆ}{\lambda }=0.272386\;(0.0425803)$

IR	-296.168	594.335	594.469	595.801	594.821	61.2883	6.24488	0.734031	<0.00001	$\overset{ˆ}{\theta }=691.666\;(122.27)$

MIR	-227.394	458.789	459.202	461.72	459.76	4.85198	1.00373	0.325486	0.002272	$\overset{ˆ}{\theta }=0.000185\;(171.337)$
										$\overset{ˆ}{\alpha }=67.9624\;(13.0293)$

OLR	-222.55	449.099	449.513	452.031	450.071	188.475	10.6229	0.999926	<0.00001	$\overset{ˆ}{\alpha }=79703\;(773090)$
										$\overset{ˆ}{\theta }=1.86346\times 10^{-10}\;(1.80747\times 10^{-9})$

R	-222.549	447.099	447.232	448.564	447.585	5.43996	0.665864	0.30478	0.00523774	$\overset{ˆ}{b}=259.457\;22.9329()$

TIR	-282.268	568.536	568.95	571.468	569.508	47.9204	5.37805	0.68194	<0.00001	$\overset{ˆ}{\theta }=617.054\;(110.03)$
										$\overset{ˆ}{\lambda }=-0.921752\;(0.0764893)$

TR	-220.217	444.433	444.847	447.365	445.405	3.83013	0.430382	0.264834	0.022468	$\overset{ˆ}{\sigma }=294.447\;(34.2319)$
										$\overset{ˆ}{\lambda }=0.610082\;(0.245437)$

TPR	-222.549	449.099	449.513	452.03	450.07	5.43996	0.665864	0.30478	0.005238	$\overset{ˆ}{\lambda }=7.42746\times 10^{-6}\;(1.32082\times 10^{-6})$
										$\overset{ˆ}{\mu }=5.88417\times 10^{-20}\;(4.65308)$

WR	-235.217	476.434	477.291	480.831	477.892	4.45707	0.867872	0.279977	0.0132516	$\overset{ˆ}{\alpha }=0.496929\;(0.115618)$
										$\overset{ˆ}{\beta }=0.0704166\;(0.0228454)$
										$\overset{ˆ}{\theta }=0.00007605\;(0.0000298)$

**Figure 7:**
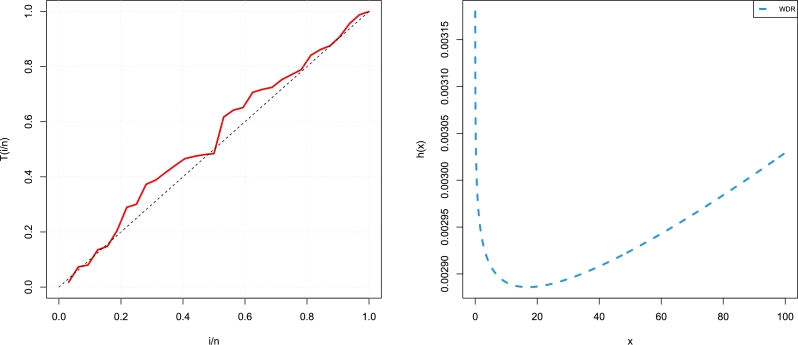
The TTT plot and fitted hrf of WDR model for the real data set.

**Figure 8:**
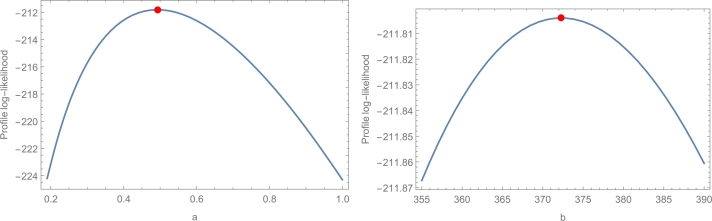
Plots of the profile-likelihood functions for the two estimated parameters.

**Figure 9:**
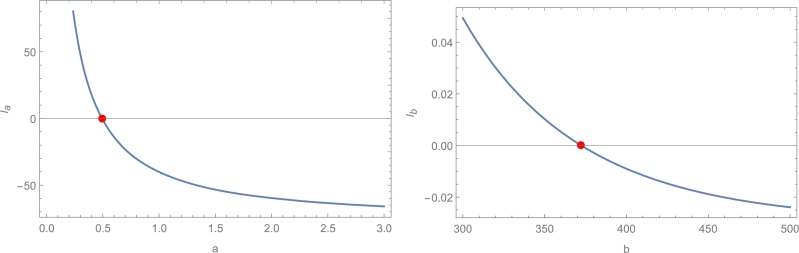
Existence and uniqueness of WDR model parameters.

**Figure 10:**
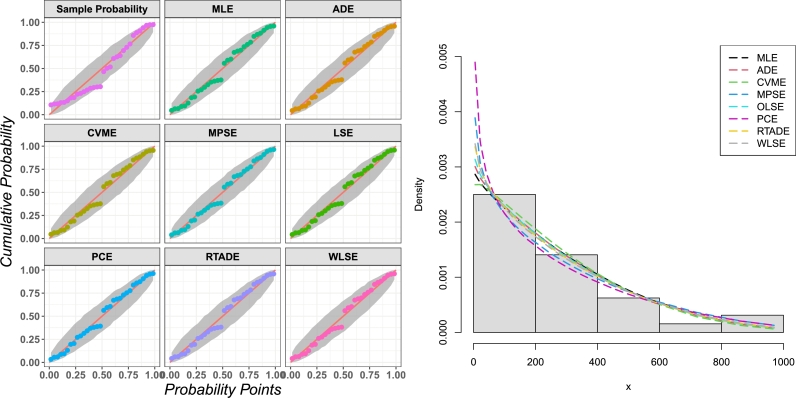
The probability-probability (PP) plot and the fitted PDFs of the proposed model for the considered real data set.

Fig. 7 presents the TTT plot with estimated HZ of WDR, Fig. 8 presents the profile of the log-likelihood function with estimated parameters, and Fig. 9 presents the uniqueness of estimated parameters of the insurance data set. Table 11 shows the values of the suggested model's estimates, log-likelihood function, CM, AD, and KS, and the KS *p*-value for each of the eight possible estimation approaches. Fig. 10 displays P-P plots for the proposed model using different estimation methods and fitted PDFs by the results of these methods.

**Table 11:** The estimates and log-likelihood function of the proposed distribution parameters and goodness-of-fit measures for the real data set by different estimation methods.

	$\overset{ˆ}{a}$	$\overset{ˆ}{b}$	L	AD	CM	KS	KSP
MLE	0.492454	372.29	-211.804	0.166395	0.0265833	0.0973316	0.922223
ADE	0.48745	367.687	-211.813	0.158638	0.0238941	0.0891767	0.961021
CVME	0.511145	348.403	-211.897	0.175449	0.0221583	0.0898852	0.958269
MPSE	0.433847	421.75	-212.126	0.249979	0.0423604	0.0860887	0.971661
OLSE	0.480932	369.364	-211.821	0.160111	0.0242457	0.0853348	0.973932
PCE	0.397801	433.433	-212.471	0.349489	0.0572919	0.102107	0.892475
RTADE	0.468065	377.614	-211.841	0.166953	0.0263597	0.081788	0.982986
WLSE	0.463306	385.058	-211.856	0.171552	0.027955	0.0840024	0.977642

## 7 The WDR distribution regression model

We can create a quantile regression model using the re-parametrized PDF (3) as presented in Bayes et al. [16], and Mitnik and Baek [30]. We will re-parametrize Equation (4) in terms of u quantile, resulting in the following:

$$b=\frac{u}{\sqrt{\log\left(\frac{1}{{\left(1-{\left(2^{p}-1\right)}^{1/a}\right)}^{2}}\right)}},\;0<p<1.$$

With this re-parametrization, the PDF and CDF of the WDR distribution are as follows:

$$F(y)=\frac{\log\left[{\left(1-{\left\{\left[1-{\left(2^{p}-1\right)}^{1/a}\right]\right\}}^{\frac{y^{2}}{u^{2}}}\right)}^{a}+1\right]}{\log(2)}$$

and

(11) $$f(y)=\frac{\log\left(\frac{1}{{\left[1-{\left(2^{p}-1\right)}^{1/a}\right]}^{2}}\right)}{u^{2}}\frac{ay{\left\{\left[1-{\left(2^{p}-1\right)}^{1/a}\right]\right\}}^{\frac{y^{2}}{u^{2}}}{\left(1-{\left\{\left[1-{\left(2^{p}-1\right)}^{1/a}\right]\right\}}^{\frac{y^{2}}{u^{2}}}\right)}^{a-1}}{b^{2}\log(2)\left[{\left(1-{\left\{\left[1-{\left(2^{p}-1\right)}^{1/a}\right]\right\}}^{\frac{y^{2}}{u^{2}}}\right)}^{a}+1\right]}.$$

Let $Y_{1},\ldots ,Y_{n}$ be n independent random variables, with each $Y_{i},i=1,\ldots ,n$ following the PDF in Equation (11) with unknown quantile parameter $u_{i}$, unknown parameters θ,a, and p belonging to the interval (0, 1) presumed to be known, that is, $Y_{i}∼WDR(a,u_{i},p)$. The WDR quantile regression model is specified here by requiring that the quantile $u_{i}$ of $Y_{i}$ satisfy the functional relationship:

$$g(u_{i})=B^{T}X_{i},\;\;i=1,\ldots ,n$$

where $B={\left(B_{0},\ldots ,B_{k-1}\right)}^{T}$ is a k-dimensional vector of unknown regression coefficients (k<n) and $x_{i}={\left(1,x_{i1},\ldots ,x_{i(k-1)}\right)}^{T}$ denotes the observations on k known covariates. We will suppose that the quantile link function g(.) maps (0, 1) into R and is strictly growing and twice differentiable. There are numerous options for link function g(.). The most well-known link functions, for example, are:

• logit: $g(u_{i})=log\left(\frac{u_{i}}{1-u_{i}}\right)$.
• probit: $g(u_{i})=\Phi^{-1}(u_{i})$, where $\Phi^{-1}(.)$ is the standard normal quantile function.
• complementary $log-log:g(u_{i})=log[-log(1-u_{i})]$.

We only consider the logit connection in this paper due to the direct interpretation of the parameters in terms of odds. Ferrari and Cribari-Neto [19] give their interpretation when $u_{i}$ is the Beta distribution's mean. When $u_{i}$ is the $q^{th}$ quantile, 0<p<1, the meanings are simple. A strictly positive link function linking the shape parameter *B* to variables $x_{i}$, which need not be equal to $x_{i}$, can also be explored. Then the quantile $u_{i}$ of $Y_{i}$ satisfies the functional relationship:

$$u_{i}=\frac{e^{B^{T}X_{i}}}{1+e^{B^{T}X_{i}}},\;\;i=1,\ldots ,n,$$

under the logit link function.

For given p=0.5 as median regression, let $\Omega =(a,B^{T})$ be estimated using the maximum likelihood method. Using the form of the PDF in Equation (11), the log-likelihood function is given by

$$l(\Omega )=n\log\left\{-2\log\left[1-{\left(2^{p}-1\right)}^{1/a}\right]+\log(a)-2\log(b)\right\}+\sum_{i=1}^{n}\log(y_{i})+\sum_{i=1}^{n}\frac{y_{i}^{2}}{u_{i}^{2}}\log\left[1-{\left(2^{p}-1\right)}^{1/a}\right]+(a-1)\sum_{i=1}^{n}\log\left(1-{\left\{\left[1-{\left(2^{p}-1\right)}^{1/a}\right]\right\}}^{\frac{y_{i}^{2}}{u_{i}^{2}}}\right)-2\sum_{i=1}^{n}\log(u_{i})-n\log\left[\log(2)\right]-\sum_{i=1}^{n}\log\left[{\left(1-{\left\{\left[1-{\left(2^{p}-1\right)}^{1/a}\right]\right\}}^{\frac{y_{i}^{2}}{u_{i}^{2}}}\right)}^{a}+1\right].$$

The MLE of parameters can't be determined analytically, hence it has to be done numerically with a Newton–Raphson or quasi-Newton optimization procedure. The asymptotic distribution of the MLE with mean vector and variance-covariance matrix V(Ω) is approximately multivariate normal under regularity conditions when n is large. The expected Fisher information matrix is

$$V(\Omega )={\left[E\left(-\frac{\partial l(\Omega )}{\partial \Omega^{2}}\right)\right]}^{-1}.$$

Simulation studies are carried out in this part to assess the finite sample behavior of the MLEs and the length of confidence intervals (Confidence level is 95%) of the parameters of the WDR model quantile regression model. For asymptotic confidence interval and bootstrapping p and t as LBP and LBT, respectively, the estimated bias, estimated average bias, mean squared error (MSE), and length of confidence interval (LCI) were obtained. All simulations were run in R using the Newton–Raphson algorithm in the “maxLik” package to get the maximum likelihood estimates.

A Monte Carlo experiment is carried out by taking

$$logit(u_{i})=B_{0}+B_{1}X_{i1}+B_{2}X_{i2},\;\;i=1,\ldots ,n,$$

where the true values of the parameters of explanatory variables (PEV) are changed as PEV I is $B_{0}$=1, $B_{1}$=0.5, and $B_{2}$=0.4. PEV II is $B_{0}$=-0.5, $B_{1}$=2.0, and $B_{2}$=1.6. PEV III is $B_{0}$=0.5, $B_{1}$=4.5, and $B_{2}$=-2. For n = 70, 150, and 300, the explanatory variables were generated from a uniform distribution and kept constant throughout the simulations. The Monte Carlo experiment was run M = 10,000 times for each scenario. The Simulation results have been shown in Table 14.

In regression application of real data: We used motor trend car road test data obtained by Harold and Paul [21]. The Motor Trend US magazine data comprised fuel consumption and ten aspects of automobile design and performance for 32 automobiles (1973–1974 models). Table 12 shows the estimates for the dependent variable by WDR distribution. Table 13 shows the estimates of the coefficient parameter of the regression model, where the independent variable is Weight (1000 lbs) and the dependent variable is Miles/(US) gallon.

**Table 12:** MLE with SE and different measures.

	a	b	KS	P-Value	AIC	CAIC	BIC	HQIC
estimates	4.1955	10.6672	0.0824	0.9815	205.6764	206.0902	208.6079	206.6481
SE	1.2443	0.9913

**Table 13:** Likelihood estimates for parameters of Regression model.

	a	*B*_0_	*B*_1_	AIC	CAIC	BIC	HQIC
estimates	0.1230	6.0063	17.0305	323.0459	323.9031	327.4432	324.5035
SE	0.0072	0.7707	0.4628

**Table 14:** MLE of regression model parameters with bootstrap confidence intervals.

PEV	n	a	0.5					1.5				
			Bias	MSE	LCI	LBP	LBT	Bias	MSE	LCI	LBP	LBT
I	70	a	0.0144	0.0050	0.2729	0.0084	0.0084	0.0083	0.0044	0.2592	0.0078	0.0079
		*B*_0_	-0.0043	0.8496	3.6149	0.1141	0.1152	-0.0277	0.9727	3.8665	0.1177	0.1172
		*B*_1_	0.0769	1.5300	4.8419	0.1607	0.1636	0.0733	1.7388	5.1636	0.1602	0.1608
		*B*_2_	0.0784	1.7568	5.1893	0.1559	0.1557	0.1109	2.0813	5.6413	0.1791	0.1782
	150	a	0.0069	0.0019	0.1697	0.0054	0.0054	0.0036	0.0018	0.1639	0.0052	0.0052
		*B*_0_	-0.0370	0.5059	2.7857	0.0938	0.0914	-0.0392	0.7238	3.3331	0.1092	0.1083
		*B*_1_	0.0798	1.0572	4.0205	0.1360	0.1350	0.1359	1.6730	5.0448	0.1648	0.1648
		*B*_2_	0.1225	1.0206	3.9329	0.1209	0.1199	0.1343	1.4127	4.6317	0.1508	0.1503
	300	a	0.0044	0.0009	0.1187	0.0038	0.0038	0.0027	0.0009	0.1143	0.0037	0.0037
		*B*_0_	0.0030	0.1602	1.5698	0.0464	0.0465	0.0063	0.1913	1.7151	0.0516	0.0509
		*B*_1_	0.0264	0.3446	2.3001	0.0753	0.0756	0.0134	0.4029	2.4889	0.0776	0.0787
		*B*_2_	0.0242	0.3859	2.4345	0.0786	0.0790	0.0207	0.4134	2.5203	0.0821	0.0820

II	70	a	0.0171	0.0052	0.2757	0.0084	0.0085	0.1009	0.0846	1.0696	0.0330	0.0329
		*B*_0_	-0.0955	0.4102	2.4839	0.0794	0.0800	-0.0439	0.2155	1.8126	0.0579	0.0576
		*B*_1_	0.1629	1.5848	4.8959	0.1548	0.1549	0.1386	1.0088	3.9015	0.1221	0.1192
		*B*_2_	0.1277	1.6444	5.0043	0.1571	0.1570	0.0877	0.8924	3.6890	0.1168	0.1170
	150	a	0.0073	0.0019	0.1671	0.0053	0.0053	0.0415	0.0278	0.6339	0.0199	0.0197
		*B*_0_	-0.0899	0.2426	1.8995	0.0604	0.0604	-0.0410	0.1034	1.2511	0.0415	0.0411
		*B*_1_	0.1408	1.1230	4.1192	0.1341	0.1329	0.0811	0.5160	2.7993	0.0923	0.0921
		*B*_2_	0.1511	0.8519	3.5711	0.1124	0.1121	0.0794	0.3460	2.2859	0.0735	0.0738
	300	a	0.0049	0.0009	0.1170	0.0037	0.0037	0.0262	0.0139	0.4507	0.0141	0.0139
		*B*_0_	-0.0148	0.0880	1.1622	0.0352	0.0348	-0.0063	0.0464	0.8443	0.0262	0.0261
		*B*_1_	0.0433	0.3145	2.1930	0.0666	0.0661	0.0304	0.1685	1.6054	0.0504	0.0508
		*B*_2_	0.0287	0.3421	2.2913	0.0746	0.0741	0.0201	0.1747	1.6373	0.0534	0.0532

III	70	a	0.0142	0.0044	0.2539	0.0079	0.0078	0.0986	0.0832	1.0632	0.0324	0.0320
		*B*_0_	-0.0438	0.6400	3.1328	0.0996	0.0996	0.0493	0.4111	2.5072	0.0787	0.0787
		*B*_1_	0.0742	1.9478	5.4660	0.1735	0.1736	0.2709	3.1087	6.8329	0.2256	0.2280
		*B*_2_	-0.0528	1.2403	4.3629	0.1343	0.1338	-0.1495	1.0143	3.9061	0.1206	0.1195
	150	a	0.0067	0.0017	0.1609	0.0051	0.0052	0.0406	0.0274	0.6299	0.0201	0.0199
		*B*_0_	-0.0402	0.3440	2.2950	0.0737	0.0727	-0.0152	0.1469	1.5018	0.0476	0.0474
		*B*_1_	0.1976	1.2336	4.2865	0.1376	0.1377	0.1471	0.7628	3.3764	0.1063	0.1065
		*B*_2_	-0.0263	0.5661	2.9490	0.0976	0.0976	-0.0146	0.2344	1.8980	0.0621	0.0630
	300	a	0.0041	0.0008	0.1105	0.0035	0.0035	0.0252	0.0137	0.4482	0.0143	0.0143
		*B*_0_	-0.0001	0.1329	1.4297	0.0452	0.0452	0.0095	0.0704	1.0401	0.0314	0.0314
		*B*_1_	0.0288	0.3955	2.4640	0.0783	0.0795	0.0489	0.3745	2.3925	0.0739	0.0753
		*B*_2_	-0.0223	0.2612	2.0024	0.0606	0.0604	-0.0219	0.1446	1.4889	0.0456	0.0466

## 8 Conclusion

This study introduces and extensively analyzes the WDR distribution, a novel two-parameter probability model that is an extended version of the classical R distribution. We have derived several fundamental distributional properties of the WDR model. We have evaluated and compared eight estimation techniques to estimate the parameters of the WDR distribution. The simulation results demonstrate that the MLE and MPSE approaches outperform the other estimation methods regarding statistical efficiency and asymptotic behavior. The heavy-tailed characteristics of the WDR distribution are further explored by calculating four risk measures: VaR, TVaR, TVP, and ES. These risk metrics highlight the distribution's usefulness in modeling extreme events and quantifying financial and actuarial risks. Additionally, we have developed a comprehensive WDR regression model and fitted it to various empirical data. A detailed case study involving an insurance lifetime data set demonstrates the superior performance of the WDR distribution compared to several competing models, including the exponential, Rayleigh, and other extended distributions such as the ABR, ExIR, ExR, GR, HLIR, IR, MIR, OLR, R, TIR, TR, TPR, and WR distributions. The findings of this study contribute to the theoretical advancement of probability distributions and have important practical implications for applied researchers and practitioners in actuarial science, risk management, and reliability engineering. The versatility and analytical tractability of the WDR distribution position it as a valuable addition to the toolbox of probability models, with the potential to enhance the modeling and analysis of a wide range of real-world phenomena.

Our proposed model may be expanded using neutrosophic statistics in future research, and we'll use the preceding publications as sources and guides in our future studies. Also, it can be applied to the censored sample method to produce random censored samples. Our research may be expanded to include applying the suggested model to several kinds of accelerated life testing.

## CRediT authorship contribution statement

**Ahmed M. Gemeay:** Writing – review & editing, Writing – original draft, Visualization, Supervision, Software, Methodology, Investigation, Formal analysis, Data curation, Conceptualization. **Eslam Hussam:** Writing – review & editing, Writing – original draft, Visualization, Validation, Supervision, Investigation. **Ehab M. Almetwally:** Writing – review & editing, Validation, Software, Resources, Methodology, Investigation, Conceptualization.

## Declaration of Competing Interest

The authors declare that they have no known competing financial interests or personal relationships that could have appeared to influence the work reported in this paper.

## Data Availability

The data that supports the findings of this study are available within the article.

## References

[1] Afify A.Z., Gemeay A.M., Ibrahim N.A. (2020). The heavy-tailed exponential distribution: risk measures, estimation, and application to actuarial data. Mathematics.

[2] Ahmad A., Ahmad S.P., Ahmed A. (2014). Transmuted inverse Rayleigh distribution: a generalization of the inverse Rayleigh distribution. Math. Theory Model..

[3] Ahmad A., Ahmad S.P., Ahmed A. (2016). Length-biased weighted Lomax distribution: statistical properties and application. Pak. J. Stat. Oper. Res..

[4] Ahmad A., Alsadat N., Atchade M.N., ul Ain S.Q., Gemeay A.M., Meraou M.A., Almetwally E.M., Hossain Md.M., Hussam E. (2023). New hyperbolic sine-generator with an example of Rayleigh distribution: simulation and data analysis in industry. Alex. Eng. J..

[5] Ahmed A., Ahmad A., Tripathi R. (2021). Topp-Leone power Rayleigh distribution with properties and application in engineering science. Adv. Appl. Math. Sci..

[6] Almarashi A.M., Badr M.M., Elgarhy M., Jamal F., Chesneau C. (2020). Statistical inference of the half-logistic inverse Rayleigh distribution. Entropy.

[7] Almetwally Ehab M., Dey Sanku, Nadarajah Saralees (2023). An overview of discrete distributions in modelling covid-19 data sets. Sankhya A.

[8] Almongy H.M., Almetwally E.M., Aljohani H.M., Alghamdi A.S., Hafez E.H. (2021). A new extended Rayleigh distribution with applications of covid-19 data. Results Phys..

[9] Almuqrin M.A., Almutlak S.A., Gemeay A.M., Almetwally E.M., Karakaya K., Makumi N., Hussam E., Aldallal R. (2023). Weighted power Maxwell distribution: statistical inference and covid-19 applications. PLoS ONE.

[10] Artzner P. (1999). Application of coherent risk measures to capital requirements in insurance. N. Am. Actuar. J..

[11] Ateeq K., Qasim T.B., Alvi A.R. (2019). An extension of Rayleigh distribution and applications. Cogent Math. Stat..

[12] Badmus N.I., Bamiduro A.T., Rauf-Animasaun M.A., Akingbade A.A. (2015). Beta weighted exponential distribution: theory and application. Int. J. Math. Sci. Optim.: Theory Appl..

[13] Bakouch H., Chesneau C., Enany M. (2020). A weighted general family of distributions: theory and practice. Comput. Math. Methods.

[14] Bashir S., Rasul M. (2018). A new weighted Rayleigh distribution: properties and applications on lifetime time data. Open J. Stat..

[15] Bashir Shakila, Masood Bushra, Al-Essa Laila A., Sanaullah Aamir, Saleem Iram (2024). Properties, quantile regression, and application of bounded exponentiated Weibull distribution to covid-19 data of mortality and survival rates. Sci. Rep..

[16] Bayes C.L., Bazán J.L., De Castro M. (2017). A quantile parametric mixed regression model for bounded response variables. Stat. Interface.

[17] Dey S., Dey T., Anis M.Z. (2015). Weighted Weibull distribution: properties and estimation. J. Stat. Theory Pract..

[18] Dey S., Dey T., Kundu D. (2014). Two-parameter Rayleigh distribution: different methods of estimation. Am. J. Math. Manag. Sci..

[19] Ferrari S., Cribari-Neto F. (2004). Beta regression for modelling rates and proportions. J. Appl. Stat..

[20] Fisher Ronald A. (1934). The effect of methods of ascertainment upon the estimation of frequencies. Ann. Eugen..

[21] Henderson Harold V., Velleman Paul F. (1981). Building multiple regression models interactively. Biometrics.

[22] Hogg R.V., Klugman S.A. (2009).

[23] Ieren T.G., Abdulkadir S.S., Issa A.A. (2020). Odd Lindley-Rayleigh distribution: its properties and applications to simulated and real life datasets. J. Adv. Math. Comput. Sci..

[24] Khan M.S. (2014). Modified inverse Rayleigh distribution. Int. J. Comput. Appl..

[25] Landsman Z. (2010). On the tail mean variance optimal portfolio selection. Insur. Math. Econ..

[26] Merovci F. (2013). Transmuted Rayleigh distribution. Austrian J. Stat..

[27] Merovci F., Elbatal I. (2015). Weibull Rayleigh distribution: theory and applications. Appl. Math. Inf. Sci..

[28] Mildenhall S.J. (1999). A systematic relationship between minimum bias and generalized linear models. Proc. Casualty Actuar. Soc..

[29] Miranda-Soberanis V.F., Yee Thomas W. (2023). Two-parameter link functions, with applications to negative binomial, Weibull and quantile regression. Comput. Stat..

[30] Mitnik P.A., Baek S. (2013). The Kumaraswamy distribution: median-dispersion re-parameterizations for regression modeling and simulation-based estimation. Stat. Pap..

[31] Nanda A.K., Jain K. (1999). Some weighted distribution results on univariate and bivariate cases. J. Stat. Plan. Inference.

[32] Olayode F. (2019). The Topp-Leone Rayleigh distribution with application. Am. J. Math. Stat..

[33] Park J.H. (1961). Moments of the generalized Rayleigh distribution. Q. Appl. Math..

[34] Qi Y. (2010). On the tail index of a heavy tailed distribution. Ann. Inst. Stat. Math..

[35] Rao C. Radhakrishna (1965). On discrete distributions arising out of methods of ascertainment. Sankhya, Ser. A.

[36] Rao C.R., Atkinson A.C., Fienberg S.E. (1985). A Celebration of Statistics.

[37] Rao G.S., Mbwambo S. (2019). Exponentiated inverse Rayleigh distribution and an application to coating weights of iron sheets data. J. Probab. Stat..

[38] Rashwan N.I. (2013). A length-biased version of the generalized gamma distribution. Adv. Appl. Stat..

[39] Reyad M.H., Hashish M.A., Othman A.S., Allam A.S. (2017). The length-biased weighted Frechet distribution: properties and estimation. Int. J. Stat. Appl. Math..

[40] Rodrigues Gabriela M., Ortega Edwin M.M., Cordeiro Gauss M., Vila Roberto (2023). Quantile regression with a new exponentiated odd log-logistic Weibull distribution. Mathematics.

[41] Roy D. (2004). Discrete Rayleigh distribution. IEEE Trans. Reliab..

[42] Saghir A., Hamedani G.G., Tazeem S., Khadim A. (2017). Weighted distributions: a brief review, perspective and characterizations. Int. J. Stat. Probab..

[43] Salama Marwa Mohamed, El-Sherpieny El-Sayed Ahmed, Abd-elaziz Abd-eltawab Ahmed (2023). The length-biased weighted exponentiated inverted exponential distribution: properties and estimation. Comput. J. Math. Stat. Sci..

[44] Sarti A., Corsi C., Mazzini E., Lamberti C. (2005). Maximum likelihood segmentation of ultrasound images with Rayleigh distribution. IEEE Trans. Ultrason. Ferroelectr. Freq. Control.

[45] Surles J.G., Padgett W.J. (2001). Inference for reliability and stress-strength for a scaled Burr type x distribution. Lifetime Data Anal..

[46] Teamah A.A.M., Elbanna A.A., Gemeay A.M. (2021). Heavy-tailed log-logistic distribution: properties, risk measures and applications. Stat. Optim. Inf. Comput..

[47] Vodă V.Gh. (1976). Inferential procedures on a generalized Rayleigh variate. I. Apl. Mat..

[48] Zelen M. (1974). Problems in cell kinetics and the early detection of disease. Reliab. Biometry.

